# DFTB Parametrization at the Example of Platinum—Implementation, Validation and Practical Considerations

**DOI:** 10.1002/jcc.70342

**Published:** 2026-03-11

**Authors:** Felix R. S. Purtscher, Armin Penz, Josef M. Gallmetzer, Jakob Gamper, Thomas S. Hofer

**Affiliations:** ^1^ Institute of General, Inorganic and Theoretical Chemistry Center for Chemistry and Biomedicine, University of Innsbruck Innsbruck Austria

## Abstract

Practical considerations for the parametrization of the transition metal platinum within the third‐order density‐functional tight‐binding (DFTB3) method are presented, enabling straightforward parametrizations of interactions between Pt and elements from the s‐, p‐, and d‐blocks of the periodic table. The newly developed parameter set is fully compatible with the 3ob DFTB3 framework, thereby extending the chemical space accessible to DFTB and enabling rapid and reliable simulations of platinum‐containing systems. The parameters were initially benchmarked against more than 1300 Pt‐containing structures extracted from the Cambridge Crystallographic Data Centre, as well as over 50 reference systems optimized at the MP2/cc‐pVTZ level of theory. Further validation included a challenging binuclear platinum(II) complex, QM/MM molecular dynamics (MD) simulations of Pt(II) complexes in aqueous solution, and 3d‐periodic DFTB‐based molecular dynamics simulations of cisplatin embedded in metal‐organic framework (MOF) hosts. Analysis of the resulting trajectories demonstrates a robust and consistent description of platinum coordination environments. To facilitate reproducibility and adoption, example Python scripts covering each step of the parametrization workflow are provided as part of the Supporting Information.

## Graphical abstract

Development and comprehensive validation of 3ob‐compatible DFTB3 parameters for platinum, enabling efficient and reliable simulations of a wide range of Pt‐containing systems.

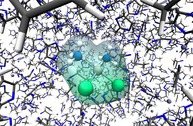

## 1 Introduction

Semi‐empirical methods such as PM7 [1], DFTB [2], and xTB [3, 4] have gained considerable attention as their performance, accuracy, and overall reliability have markedly improved over the past three decades. These approaches aim to reduce computational cost by employing well‐validated parameters while retaining much of the accuracy associated with quantum‐chemical methods, including Hartree‐Fock theory, density functional theory (DFT), and post‐Hartree‐Fock methods. Most semi‐empirical methods treat only the valence electrons explicitly, using pseudopotentials to account for core electrons. However, the parameterization process for density‐functional tight binding (DFTB) remains challenging and labor‐intensive, particularly for transition metals such as iron [5], zirconium [6, 7], and platinum, which are of key importance in many areas of research.

Platinum complexes play a central role in a wide range of chemical and biomedical applications, with distinct functionalities arising from the respective oxidation state. Platinum(II) complexes are widely known for their square‐planar geometry and favorable ligand‐exchange kinetics, and are widely employed in homogeneous catalysis [8, 9], photoemission [10] and supramolecular assembly [11]. In addition, they constitute the foundation of clinically established anticancer drugs such as cisplatin, carboplatin, and oxaliplatin [12]. Beyond potential medical applications [13, 14, 15], Pt(IV) complexes are increasingly explored as catalysts [16, 17], photoemissive materials [18] as well as precursors for thin‐film deposition [19]. Together, the complementary properties of Pt(II) and Pt(IV) complexes form the foundation for their extensive applications in various areas, including catalysis, coordination chemistry, materials development, and medicinal chemistry.

In the latter field in particular, platinum‐based complexes remain central to modern cancer chemotherapy owing to their proven efficacy across a wide spectrum of malignancies [20]. Cisplatin represents the prototypical agent in this class and exerts its cytotoxicity primarily through the formation of DNA adducts that disrupt replication and transcription, ultimately triggering apoptosis. Although cisplatin and its clinical successors are highly effective, their use is limited by severe dose‐dependent toxicities and the emergence of resistance. These challenges have stimulated extensive efforts to design next‐generation platinum compounds with improved therapeutic profiles. Approaches include structural modification of Pt(II) and Pt(IV) scaffolds to enhance selectivity [21, 22], platinum‐containing nanoparticles [23], as well as nanomaterial‐based delivery systems that enable targeted accumulation and reduced off‐target damage [24, 25].

In the latter context, metal‐organic frameworks (MOFs) have emerged as a highly promising class of drug‐delivery compounds owing to their structurally versatile and chemically tunable architectures [25, 26, 27, 28]. Through controlled modulation of pore size, topology, and surface functionality, MOFs can be precisely tailored to accommodate specific physicochemical requirements of therapeutic compounds [29, 30]. Such tunability enables the encapsulation of a broad spectrum of active molecules while maintaining them in a chemically favorable environment.

In addition to their structural adaptability, many MOFs exhibit excellent biocompatibility and biodegradability, and their drug‐loading capacity, retention behavior, and release profiles can be finely adjusted through the choice of metal nodes, linkers, and framework stability. The pH‐responsive lability of certain systems, such as ZIF‐8, enables site‐specific release in acidic environments [31, 32], thereby enhancing therapeutic efficiency while reducing off‐target toxicity to surrounding healthy tissue. Moreover, MOFs can improve the solubility and stability of otherwise challenging drug molecules, broadening the scope of viable therapeutic cargo [33].

Despite these advantages, a detailed molecular‐level understanding of host‐guest interactions remains a major challenge for the rational design of drug@MOF systems. Recent studies have shown that the DFTB method, when combined with molecular dynamics (MD) simulations, offers a robust computational framework for investigating both pristine MOFs [34, 35] as well as guest@MOF systems [36, 37, 38, 39, 40]. Applications include gas storage [37, 38] and the incorporation of small organic molecules [36, 40] within different frameworks, demonstrating the method's ability to capture structure, stability, and dynamic behavior. In contrast to static structure optimizations, MD explicitly treats thermal motion and time evolution, thereby providing access to phenomena such as diffusion [37, 38], host‐induced conformational changes [39], and competitive interactions including guest‐guest stacking [40].

Although the platinum parameters developed in this work are broadly applicable and not restricted to drug‐delivery systems, their primary motivation was to enable accurate DFTB simulations of Pt‐based therapeutics embedded in MOF hosts. To this end, a consistent set of third‐order DFTB parameters for platinum, together with associated pair‐wise repulsive potentials for Pt‐{H, C, N, O, F, Cl, Na, Mg, Zn, Zr} interactions, has been generated. This parameter set substantially expands the chemical space accessible to DFTB and enables reliable simulations of a wide range of Pt‐containing compounds relevant to drug delivery and beyond.

## 2 Methods

This section focuses on the methods and software used to generate the different parameters. DFTB parameters can be divided in three categories [2, 41]: (i) one‐center parameters, derived for each specific element, (ii) Slater‐Koster tables for element‐element pairs containing the precomputed Hamiltonian and overlap matrix elements evaluated using DFT functionals (e.g., LDA or PBE [42]) and (iii) the force field‐like repulsive potential, again formulated for a specific element‐element pair. The total energy expression for the self‐consistent charge (SCC) electronic part (i.e., at DFTB2 level) consists of the following terms

(1) $$E_{elec}^{SCC-DFTB}=\overset{n_{e}}{\underset{i}{\sum }}\langle \psi_{i}|{Ĥ}_{0}|\psi_{i}\rangle +\frac{1}{2}\underset{\alpha \beta }{\sum }\Delta q_{\alpha }\Delta q_{\beta }\gamma_{\alpha \beta }$$

with ψ denoting the molecular orbitals, typical expanded in a minimial basis of confined pseudoatomic Slater‐type orbitals, $\Delta q_{\alpha }$ denotes the net charge of atom α and $\gamma_{\alpha \beta }$ is a function describing the coulombic interaction between the spherical charge distributions of atoms α and β. For large distances, this function coincides with the prototypical Coulomb interaction $r_{\alpha \beta }^{-1}$, while in the short‐range limit $r_{\alpha \beta }\to 0$ the electron‐electron interaction is expressed via the atomic Hubbard parameter $U_{\alpha }$. The latter can be shown [2, 41] to be directly proportional to the chemical hardness η via:

(2) $$U_{\alpha }=2\eta_{\alpha }$$

The associated repulsive contributions are expressed as

(3) $$E_{rep}=\underset{I<J}{\sum }V_{rep}^{IJ}(R_{IJ}).$$

In practice, $V_{rep}^{IJ}$ is represented via a short‐ranged exponential function that transitions into a set of cubic spline intervals that are determined via parameterization.

Beyond this second‐order formulation, the third‐order extension of DFTB (DFTB3) introduces an additional term in the total energy expansion that accounts for cubic fluctuations of the atomic charges [43]

(4) $$E^{\left(3\right)}=\frac{1}{3}\underset{\alpha \beta }{\sum }\Delta q_{\alpha }^{2}\Delta q_{\beta }\Gamma_{\alpha \beta }$$

with $\Gamma_{\alpha \beta }$ representing the derivative of $\gamma_{\alpha \beta }$ with respect to the atomic charge $q_{\alpha }$. This correction was shown to improve the description of charge redistribution, reduce overpolarization effects, and yield more reliable energetics for systems exhibiting strong hydrogen bonding, proton transfer, or pronounced charge separation [44]. The third‐order contribution depends on additional, element‐specific response parameters referred to as Hubbard derivatives $U^{\text{d}}$, and modifies the charge‐charge interaction by incorporating the nonlinear response of the atomic reference densities. This term augments the SCC matrix while leaving the construction of the Slater‐Koster tables and the generation of the repulsive potential unchanged, thereby retaining the overall efficiency of DFTB2 while improving accuracy for a wide variety of systems.

All of the parameters require a calculation at a higher level of theory; the procedure will be explained in detail in the following subsections. In principle, all calculations necessary to determine the parameters of the electronic part can be carried out with the Python‐based program hotcent [45, 46] interfaced to open‐source library libxc [47]. Popular DFT software could also be employed for part of the parametrization (one electron properties such as Hubbard derivatives and spin coupling constants), provided a functionality for calculations using fractional occupation numbers, for example, 0.95 instead of a full electron is implemented, as for instance available in Molpro [48, 49, 50].

### 2.1 One‐Center Parameters: Orbital Eigenvalues, Hubbard Values, Hubbard Derivatives and Spin Constants

The one‐center parameters consist of the following parts and are derived from DFT calculations of the isolated atom. This parameterization steps typically involve calculations with fractional electron occupations:

- Orbital eigenvalues: $\varepsilon_{s}$, $\varepsilon_{p}$, $\varepsilon_{d}$
- Hubbard hardness parameters: $U_{s}$, $U_{p}$, $U_{d}$
- Hubbard derivatives: $U_{s}^{d}$, $U_{p}^{d}$, $U_{d}^{d}$
- Spin constants: $W_{ss}$, $W_{pp}$, $W_{dd}$, $W_{sp}$, $W_{sd}$, $W_{pd}$

The calculation of the Hubbard derivatives is dependent on the change of the orbital energy with respect to the occupation:

(5) $$U^{d}=\frac{\epsilon_{n,h}-2\epsilon_{0}+\epsilon_{n,l}}{\Delta n^{2}}$$

Similarly, calculation of the spin constants, based on this spin‐polarized version of Janak's theorem [51], is carried out via the evaluation of the orbital energies with respect to occupation of *other* orbitals:

(6) $$W_{A,ij}=\frac{1}{2}\left(\frac{\partial \varepsilon_{i↑}}{\partial n_{j↑}}-\frac{\partial \varepsilon_{i↑}}{\partial n_{j↓}}\right)$$

For all of these parameters, respective functions for their determination are implemented in hotcent [45, 46], except for the calculation of the Hubbard derivatives, which can be easily added manually as outlined in Section S6 of the Supporting Information. For all calculations the scalar‐relativistic correction as implemented in hotcent have been applied. While in many cases the same parameter is applied to the s‐, p‐ and d‐shell, if required, the importance of angular momentum dependent values for the Hubbard parameters and the associated derivatives has been outlined by Gaus et al. in the DFTB3 parametrization for copper [52]. It should be noted that both the eigenvalues of unoccupied orbitals and Hubbard parameters along with the associated derivatives are sometimes manually adjusted to achieve increased accuracy [52].

### 2.2 Slater‐Koster Tables

Slater‐Koster tables contain the values of the shell‐resolved two‐center integrals in the form of precalculated, distance‐dependent Hamiltonian and overlap matrix elements H and S of the respective orbital combinations (i.e., $ss^{\sigma },sp^{\sigma },pp^{\sigma }$, $pp^{\pi }$, etc.). The creation of the Slater‐Koster tables is carried out based on DFT reference calculations. The use of suitably adjusted confinement potentials facilitates the truncation of the diffuse tails of the wave functions associated with the tight‐binding formalism. In general, two forms of confinement potentials are commonly employed, being the simpler quadratic form

(7) $$V_{conf}={\left(\frac{r}{r_{0}}\right)}^{2}$$

and the Woods‐Saxon confinement [53] originating from nuclear physics

(8) $$V_{conf}^{WS}=\frac{w}{1+e^{-a(r-r_{0})}},$$

each associated to respective advantages and disadvantages. A comparison of the characteristic forms of the quadratic and Woods‐Saxon confinement is depicted in Supporting Information, Figure S1.

In case of the extension of an already existing DFTB parameterization (in this case the 3ob set [44, 54, 55, 56]), the same approach has to be utilized. Thus, in the present work a quadratic confinement had to be applied to ensure consistency across the parameter set. In order to provide an adequate reference for the determination of the confinement parameters of Pt, an electronic band‐structure calculation of Pt(fcc) has been carried out at PBE level of theory [42] in conjunction with the pob‐TZVP‐rev2 basis set [57] using the solid‐state quantum‐chemical program Crystal23 [58]. The pob‐TZVP‐rev2 basis set applied to Pt incorporates a relativistically derived effective core potential (ECP) representing the [Kr]4$d^{10}4f^{14}$ core, thereby accounting for the dominant relativistic contraction effects in the reference calculations [59].

### 2.3 Repulsive Potentials

The repulsive potentials usually consist of an exponential function at small internuclear distances that transitions into a set of cubic spline functions to describe the interactions near the equilibrium distance and beyond. Again, the repulsive potentials are parametrized against high‐level calculations such as hybrid DFT, perturbation theory and potentially even coupled cluster calculations. In case of the 3ob set for organic molecules [44, 54], a combination of methods was employed involving optimized geometries of small neutral closed‐shell molecules obtained at B3LYP/cc‐pVTZ level, atomization energies calculated from the composite G3B3 [60] method combining B3LYP [61] and CCSD(T) [62] calculation results as well as proton transfer barriers determined at MP2/G3large (structures and energetics) level of theory. In contrast, in the extension of the 3ob parameters toward Cu the authors have shown that the choice of B3LYP/aug‐cc‐PVTZ as reference method provides a highly suitable compromise between accuracy and computational effort [52]. Finally, in the recent extension of the 3ob set by Zr reference data at the MP2/cc‐PVTZ level of theory could be applied with large success, yielding data of comparable accuracy as achieved in the previous paramatrizations [7]. For this reason, and because no predefined basis set for Pt is available for the advanced G3 composite methods, all reference calculations were performed at the MP2 level of theory, employing cc‐PVTZ‐PP for Pt [59] and Zr [63] and cc‐pVTZ basis sets for the remaining elements {C, H, N, O, F, Cl, Na, Mg, Zn} [64, 65, 66].

All MP2 and DFT reference calculations were carried out with Gaussian16 [67] and Turbomole [68, 69, 70]. In case of the Turbomole calculations the resolution‐of‐identity (RI) setting [71], employing adequate auxiliary basis sets [72], was used in order to reduce the computation time.

The repulsive fitting process was carried out with tango [73] requiring the Slater‐Koster files containing the electronic part as input. Similarly to the case of the pob‐TZVP‐rev2 basis set applied in the solid‐state calculation of Pt(fcc) the cc‐pVTZ‐PP basis incorporates a relativistic ECP to implicitly take relativistic contraction effects into account [59]. In addition, all calculations carried out in tango were performed using the same scalar‐relativistic correction as implemented in hotcent discussed above.

### 2.4 Validation

The parameters derived in this work are validated by comparison of DFTB‐optimized geometries to structures determined by experimental methods as well as RIMP2 calculations of small Pt‐containing complexes. Furthermore, sequential bond dissociation energies and proton affinities of small molecular systems evaluated by DFTB were compared to high‐level calculations (MP2). Quantum mechanical/molecular mechanic MD simulations (QM/MM MD) of platinum‐containing complexes in aqueous solution were executed to assess the performance in a dynamic environment at nonzero thermal conditions. In detail, the respective geometry optimizations of the individual species were carried out using Turbomole employing perturbation theory at the RIMP2/cc‐pVTZ level. The DFTB geometry optimizations were carried out employing DFTB+ [74] in combination with the existing 3ob parameters. For both geometry optimizations and MD simulations, the DFTB Hamiltonian was augmented with the D3 dispersion correction developed by Grimme et al. [75], along with the halogen and hydrogen corrections intrinsic to the 3ob parameterization [44], whenever the corresponding atomic species were present in the molecular structures.

QM/MM MD simulations of platinum‐containing species in aqueous environment in the canonical (*NVT*) ensemble employing the new parameters were carried out, using the simulation protocol used in previous works [76, 77]. Energy and force calculations were carried out employing the quantum mechanical charge field (QMCF) program [78, 79, 80] interfaced to the DFTB+ software [74] and internal force field routines. Thermostatisation at 298.15 K of the system was achieved by employing the Berendsen thermostat [81]. The time step was set to 0.5 fs to allow an explicit and stable representation of vibrational motion involving hydrogen‐containing degrees of freedom. The interaction between the water molecules in the MM region was evaluated utilizing the flexible BJH‐CF2 model for water [82, 83]. The respective radial distribution functions (RDFs) derived from the MD trajectories were then compared to simulation results taken from the literature [76, 77].

The cisplatin@ZIF‐8 and cisplatin@UiO‐66 simulations were carried out in the QMCF program interfaced to the DFTB+ software [74], but in contrast to the QM/MM simulations, the calculation of energy and forces was performed entirely at the DFTB3 level under periodic boundary conditions. The simulations were conducted in analogy to previous successful MD studies of guest@MOF systems using DFTB3 in conjunction with the 3ob parameter set [7, 34, 35, 36, 37, 38, 40]. The guest molecule was inserted into the pore of the preequilibrated MOF systems taken from previous studies [7, 34], ensuring a minimum distance of 2.0 Å between all atoms of the host and the guest, respectively. In the case of UiO‐66, the larger octahedral pore was selected for the guest insertion. As established in the previous studies, the SHAKE/RATTLE routines [84, 85] were employed to restrain hydrogen atoms to their respective neighboring atoms, thereby enabling access to an increased time step of 2.0 fs. In order to demonstrate that the newly developed DFTB parameters for Pt enable a stable description of the systems at nonzero thermal conditions under periodic boundary conditions, a total sampling of 50 000 MD steps (100 ps) have been carried out at 298.15K in the NPT ensemble, following an equilibration under NVT and NPT conditions, each carried out for 10 000 MD steps (20 ps).

## 3 Results

### 3.1 Electronic Part

To maintain consistency with the parametrization strategy of the 3ob set, the one‐center parameters were computed using the PBE functional [42] (in libxc notation: GGA_X_PBE + GGA_C_PBE), incorporating scalar‐relativistic corrections as implemented in hotcent [45, 46]. A Python script illustrating the generation of these parameters is provided in the Supporting Information (Listing S1), and the resulting values are summarized in Table 1. The Hubbard parameter and the corresponding derivative for the d‐orbitals $U_{\text{d}}$ and $U_{\text{d}}^{\text{d}}$ were adjusted to accurately reproduce the energetics of the reaction $\text{Pt}\to {\text{Pt}}^{2+}+2{\text{e}}^{-}$, reflecting the focus of this study on achieving a reliable description of ${\text{Pt}}^{\text{II}}$‐containing systems. The latter strategy follows the approach established in an earlier DFTB parametrization study for Cu, in which the values of $U_{\text{d}}$ and $U_{\text{d}}^{\text{d}}$ were adjusted to reproduce the experimental oxidation potential of the ${\text{Cu}}^{\text{I}}\to {\text{Cu}}^{\text{II}}+{\text{e}}^{-}$ redox couple due to its key relevance in biological applications [52].

**TABLE 1:** Electronic DFTB3 parameters (quadratic confinement potentials, orbital eigenvalues, Hubbard values and associated derivatives, spin constants) determined for platinum from DFT reference calculations carried out at PBE level using the hotcent package.

DFTB conf.	
$r_{0,sp}$	3.13
$r_{0,d}$	5.82
$r_{0,dens}$	6.91
DFTB prop.	
$\epsilon_{s}$	−0.20734
$\epsilon_{p}$	−0.03096
$\epsilon_{d}$	−0.22595
$U_{sp}$	0.2897
$U_{d}$	0.2380
$U_{sp}^{d}$	−0.03934
$U_{d}^{d}$	−0.07
$W_{A,ss}$	−0.01211
$W_{A,sp}$	−0.00804
$W_{A,sd}$	−0.00582
$W_{A,pp}$	−0.01897
$W_{A,pd}$	−0.00583
$W_{A,dd}$	−0.01032

*Note:* All values are given in atomic units unless they are unitless.

The generation of Slater‐Koster tables always has to strike a compromise between molecular and bulk structure reference systems. In this work, the band structure calculated at DFTB level was compared to a DFT calculation of fcc platinum at the PBE/pob‐TZVP‐rev2 level. First, different occupations of the s‐ and d‐orbitals have to be tested to assess the respective advantages of the two electronic configurations $5d^{9}6^{1}$ and $5d^{8}6s^{2}$. A compromise between the two electron configurations was achieved by pre‐calculating the two‐center integrals with $5d^{8}6s^{2}$, but setting the occupation to $5d^{9}6^{1}$. This configuration also provides an additional degree of freedom for tuning the Hubbard parameter and its derivative for the d‐shell. In the case of ${\text{Pt}}^{\text{II}}$, formation of the +2 oxidation state effectively involves removing one electron from the s‐shell and one from the d‐shell. Incorporating this electronic redistribution into the parametrization greatly improved the reproduction of the target reference energies.

Following the band‐structure fit used to optimize the orbital and wave‐function confinement parameters (a Python script is provided in Supplementary Listing S2), the resulting confinement settings were employed as input for a systematic scan of the density‐confinement parameter as well as the $k_{B}T$ parameter in tango [73]. Adjusting $k_{B}T$ within the range of 2 to 30 eV can improve the electronic contribution to the fit, as lower values increase the relative weight of bands located closest to the reference energies (i.e., the Fermi level or valence‐band maximum). In addition to these parameters, the orbital eigenvalues exert a critical influence on the relative placement of the electronic bands, either shifting them upward or downward in energy. Consequently, the eigenvalues must be defined prior to executing the fitting procedure. After performing an initial fit, these values can then be refined through targeted fine‐tuning.

**FIGURE 1:**
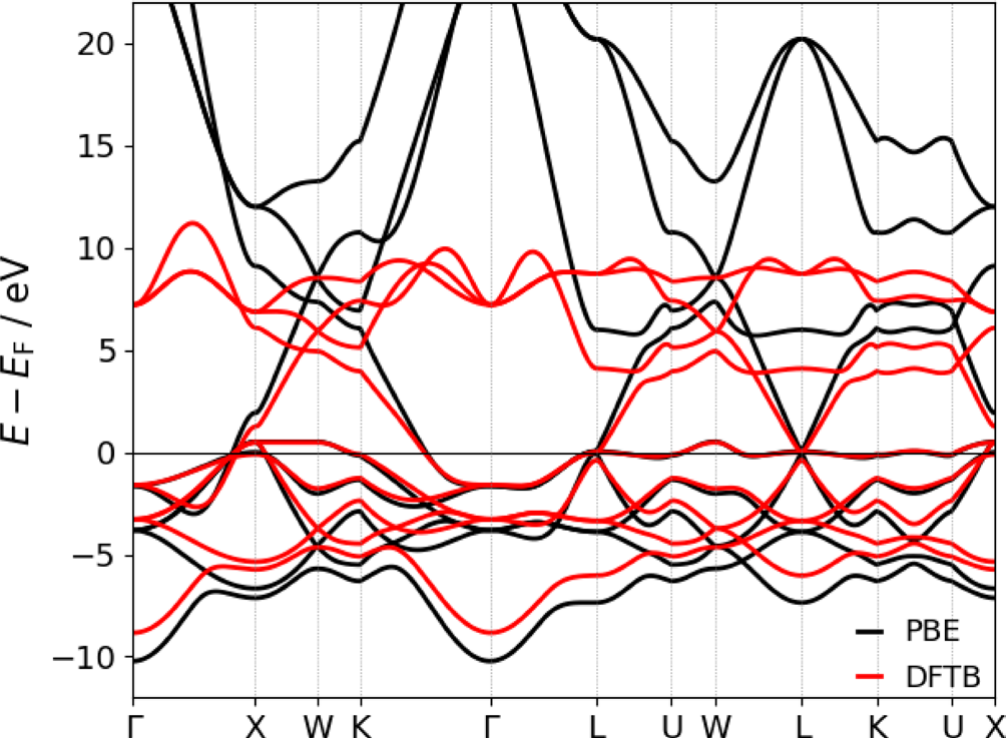
Platinum band structures of the PBE reference (black) and the DFTB calculation (red) after the fitting procedure.

The resulting band‐structure fit for the final parameterization is depicted in Figure 1. As expected, some deviations between the DFTB and DFT reference bands remain, reflecting both the compromises inherent in the Slater‐Koster table construction discussed above as well as approximations intrinsic to the DFTB method. The latter involves the use of a minimal STO‐type basis and, foremost, the specific confinement potentials employed. Electronic band structures obtained with parameters optimized primarily for molecular systems often appear compressed relative to those tailored for materials‐oriented applications. This effect is particularly pronounced for transition metals such as Cu [52] and Pt, which are significantly more sensitive to confinement potentials than main‐group elements (e.g., Mg) or electronically simpler transition metals such as Zn, whose filled 3d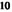 shell leads to more robust behavior.

Despite the overall compression, the highest occupied band crossing the Fermi level shows excellent agreement with the DFT reference, and only minor deviations are observed for the bands in the range from −2 to 0 eV, with larger discrepancies appearing at lower valence energies. While further tuning could improve agreement for these lower‐lying bands, such adjustments would compromise the accuracy of the key oxidation reaction $\text{Pt}\to {\text{Pt}}^{2+}+2{\text{e}}^{-}$, which was a central target of the present parametrization. Consequently, the present parameter set should not be expected to yield an accurate description of bulk Pt and Pt‐containing solid‐state systems, but represents a deliberate compromise prioritizing reliable energetics for Pt(II)‐relevant chemistry.

It is important to note that this behavior is not a limitation unique to the Pt parametrization developed in this work, but reflects a defining characteristic of the 3ob set. Previous parametrizations for Mg, Zn, Cu, and Zr [7, 52, 54] explicitly emphasize that the 3ob set is optimized for ions in solution and biologically relevant molecular systems. As a consequence, the parameters are less suited when aiming at a comprehensive reproduction of metallic band structures or the properties of metal‐containing solid‐state materials such as bulk oxides. Despite these constraints, the Pt parameters developed in this work integrate seamlessly into the existing 3ob set and substantially expand its applicability, enabling calculations across a wide variety of chemically relevant systems containing platinum, as will be demonstrated in the following sections.

### 3.2 Repulsive Potentials

All repulsive potentials were fitted against MP2/cc‐pVTZ calculations of small molecular systems in either a neutral or charged state, for example, for the Pt‐N interaction potential energy scans of ${\left[\text{Pt}({\text{NH}}_{2})\right]}^{+}$, ${\left[\text{Pt}{\left({\text{NH}}_{3}\right)}_{4}\right]}^{2+}$ and ${\left[\text{Pt}{\left({\text{NH}}_{2}\right)}_{4}\right]}^{2-}$ were carried out. The employed reference systems are listed in Table 2, an example Python script for fitting all interactions using the tango package [73] is provided in the Supporting Information (listing 5), the resulting potentials in dependence of the interatomic distance are plotted in Figures S2 and S3.

**TABLE 2:** Reference systems for parametrizing the respective repulsive interactions.

Interaction	Fit systems
Pt‐H	$\text{Pt}{\text{H}}_{2}$
Pt‐C	$\text{Pt}{\left({\text{CH}}_{3}\right)}_{2}$, ${\left[\text{Pt}({\text{CH}}_{3})\right]}^{+}$, ${\left[\text{Pt}{\left({\text{CH}}_{3}\right)}_{4}\right]}^{2-}$, ${\left[\text{Pt}(\text{CO})\right]}^{2+}$, ${\left[\text{Pt}{\left(\text{CO}\right)}_{2}\right]}^{2+}$
Pt‐N	${\left[\text{Pt}({\text{NH}}_{2})\right]}^{+}$, ${\left[\text{Pt}{\left({\text{NH}}_{3}\right)}_{4}\right]}^{2+}$, ${\left[\text{Pt}{\left({\text{NH}}_{2}\right)}_{4}\right]}^{2-}$
Pt‐O	${\left[\text{Pt}{\left({\text{H}}_{2}\text{O}\right)}_{4}\right]}^{2+}$, $\text{Pt}{\left(\text{OH}\right)}_{2}$
Pt‐F	${\text{PtF}}_{2}$, ${\left[{\text{PtF}}_{4}\right]}^{2-}$
Pt‐Cl	${\text{PtCl}}_{2}$, ${\left[{\text{PtCl}}_{4}\right]}^{2-}$
Pt‐Na	PtNa
Pt‐Mg	PtMg
Pt‐Zn	PtZn
Pt‐Zr	PtZr

All repulsive potentials show the expected exponential decay with respect to distance, although in some cases shallow minima (Pt‐{C, N, Zn}) and shoulders (Pt‐{O, F, Mg, Zr, Pt}) are visible in the region associated to the cubic spline representation (see Supporting Information, Figures S2 and S3). While this may appear as a potential short‐coming, the magnitude of these deviations remains well within the range observed for other interactions within the original 3ob set [44, 54, 55, 56] including the prominent interaction pairs {C, N, O}‐H as well as C–C and N–O (see Supporting Information, Figure S4).

### 3.3 Validation

#### 3.3.1 Structural Properties

Similar to the parametrization of Mg, Zn, Cu, and Zr in the 3ob set [7, 44, 52, 54], small coordination complexes of the respective ion served as a reference to validate the results of the repulsive fitting.

In the first step, the validation of parametrization against experimental reference structures was carried out, utilizing a systematic search of Pt‐containing compounds from the Cambridge Crystallographic Data Centre (CCDC) [86]. The structures were extracted by {C, H, N, O, Cl, F} as optional elements directly coordinated to a Pt‐central species. The extraction of the coordination complexes was carried out using the Python package MOFUN by removing counterions, solvent molecules, and other structural elements not directly involved in Pt‐coordination [87].

All extracted structures were subject to an energy minimization using the 3ob parameters, including those developed in this work. Similar to previous studies [7, 44, 52, 54] the energy minimizations were executed in absence of periodic boundary conditions before determining the deviation from reference structures via the Kabsch algorithm [88], as implemented in an open‐source Python package specifically developed for the RMSD analysis of molecular systems [89].

Evaluation of the deviations between the experimental structures and the minimized geometries revealed 46 cases in which MOFUN incorrectly extracted the coordination complex. In most instances, the presence of additional counterions led to substantial structural displacement during vacuum optimization, artificially increasing the RMSD. A few cases involved fragmented molecular structures. The RMSD values for the remaining 1324 Pt complexes were computed with hydrogen atoms omitted to prevent rotamer‐related artifacts and are reported in the Supporting Information, Listing S8.

**FIGURE 2:**
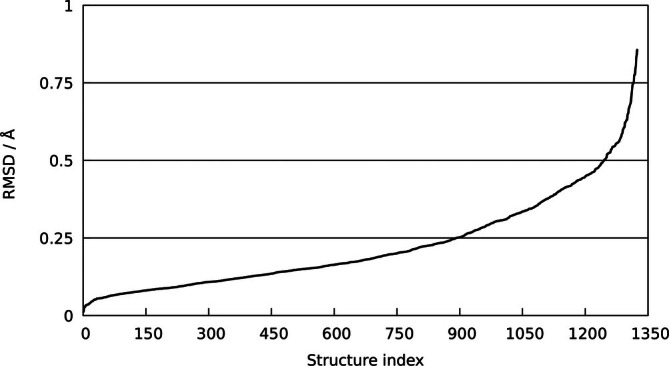
RMSD values of DFTB‐optimized structures compared to the experimental reference structures taken from the CCDC database.

Figure 2 depicts the RMSD values for the 1324 investigated compounds in ascending order. From this analysis, it can be seen that 67.1% of all evaluated structures (i.e., 891 of 1324 systems) exhibit an RMSD of ≤ 0.25 Å, 94.0% fall within ≤ 0.5 Å (i.e., 1245 of 1324 systems), and 99.3% show an RMSD of ≤ 0.75 Å (i.e., 1315 structures). In addition, the mean and maximum deviations in Pt‐{C, N, O, F, Cl} bond lengths across the dataset have been monitored (see Table 3), considering atoms within 2.5 Å as nearest neighbors.

**TABLE 3:** Number of structures $n_{\text{struct}}$, number of analyzed pair distances $n_{\text{pair}}$, mean and maximum deviations of the Pt‐{C,N,O,F,Cl} distances in Å between the DFTB‐optimized geometries and experimental reference structures taken from the CCDC database. Only atoms within a distance of 2.5 Å of the Pt atom were considered.

Bond	$n_{\text{struct}}$	$n_{\text{pair}}$	Mean	Max
Pt‐C	549	1188	0.0785	0.515
Pt‐N	1190	2639	0.0818	0.591
Pt‐O	318	577	0.0583	0.300
Pt‐F	11	15	0.0772	0.160
Pt‐Cl	792	1439	0.0325	0.281

The two structures with the largest RMSD values (CCDC identifiers CCXAPT and EKAFON) are shown in the Supporting Information, Figure S5. Given the very good agreement in the coordination environment at the metal center, these elevated RMSD values do not arise from deficiencies in the DFTB parametrization. Instead, the comparatively large and bulky ligands are no longer constrained by the crystal packing forces and undergo substantial relaxation under vacuum conditions, amplifying the structural deviations relative to the experimental geometries.

**FIGURE 3:**
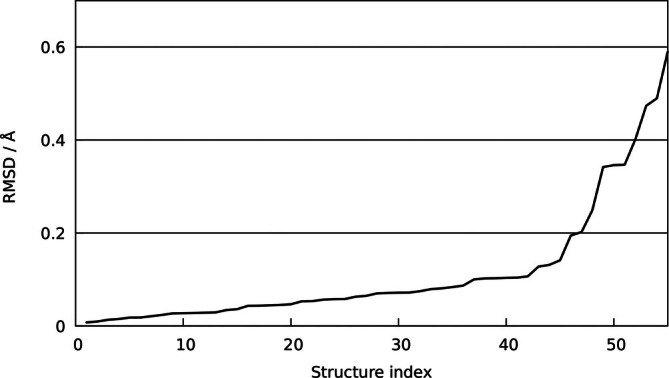
RMSD values of DFTB‐optimized structures relative to reference structures minimized at the MP2/cc‐pVTZ level of theory.

Naturally, optimizing isolated complexes in the absence of the crystal environment may lead to configurational shifts relative to the experimental structures, as any lattice‐induced strain can relax under vacuum conditions. To account for this, a smaller subset of complexes in oxidation state +2 was taken from the earlier work on Cu [52]. Since platinum(II) compounds tend to form square‐planar coordination complexes, all structures containing coordination number 5 have been excluded. The remaining 55 structures were then subject to energy minimization at MP2/cc‐pVTZ and DFTB3 levels of theory, respectively. Again the RMSD values in the absence of H‐atoms as well as the mean and maximum deviations in Pt‐{H, C, N, O, F, Cl} have been analyzed (see Figure 3 and Table 4), considering all atoms within 2.5 Å as nearest neighbors (expect for hydrogen atoms, in which case a cutoff distance of 1.75 Å was used).

**TABLE 4:** Number of structures $n_{\text{struct}}$, number of analyzed pair distances $n_{\text{pair}}$, mean and maximum deviations of the Pt‐{H,C,N,O,F,Cl} distances in Å between the DFTB‐optimized geometries and reference structures minimized at MP2/cc‐pVTZ level of theory.

Bond	$n_{\text{struct}}$	$n_{\text{pair}}$	mean	max
Pt‐H	3	2	0.0651	0.0704
Pt‐C	13	6	0.0276	0.0571
Pt‐O	51	25	0.0511	0.2092
Pt‐N	61	25	0.0538	0.2043
Pt‐F	13	6	0.0265	0.0709
Pt‐Cl	12	5	0.0190	0.0412

From the sorted RMSD plot it can be seen that 65% remain below a total RMSD of 0.1 Å, 84% are within 0.2 Å, and 93% remain below an RMSD value of 0.4 Å. The only notable outlier exhibiting an RMSD of 0.59 Å corresponds to a complex containing comparatively weakly interacting (CH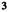)
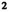
N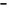 ligands in combination with an open coordination site (see Supporting Information, Figure S6). In this case, a structural relaxation occurs in which ligands in trans‐position adopt a larger angle, leading to a slight tilting in the orientation of the ligands, driven by weak dispersive interactions between the CH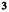 groups. It should be emphasized, however, that this deviation cannot be attributed exclusively to the newly derived Pt parameters, as contributions from other elements of the 3ob set (most prominently the N–C and C–C interactions) also play a significant role.

These results from the structural analysis clearly demonstrate that the newly derived Pt parameters reliably reproduce the geometries of a wide variety of experimentally characterized Pt‐containing complexes. Only a small number of outliers are observed, and their deviations remain well within the expected accuracy limits of a semi‐empirical method such as DFTB. It should be emphasized that any observed shortcomings arise from the interplay between parameters of different atomic species and cannot be attributed solely to the newly developed Pt parameters.

#### 3.3.2 BFPtPZ Complexes

**FIGURE 4:**
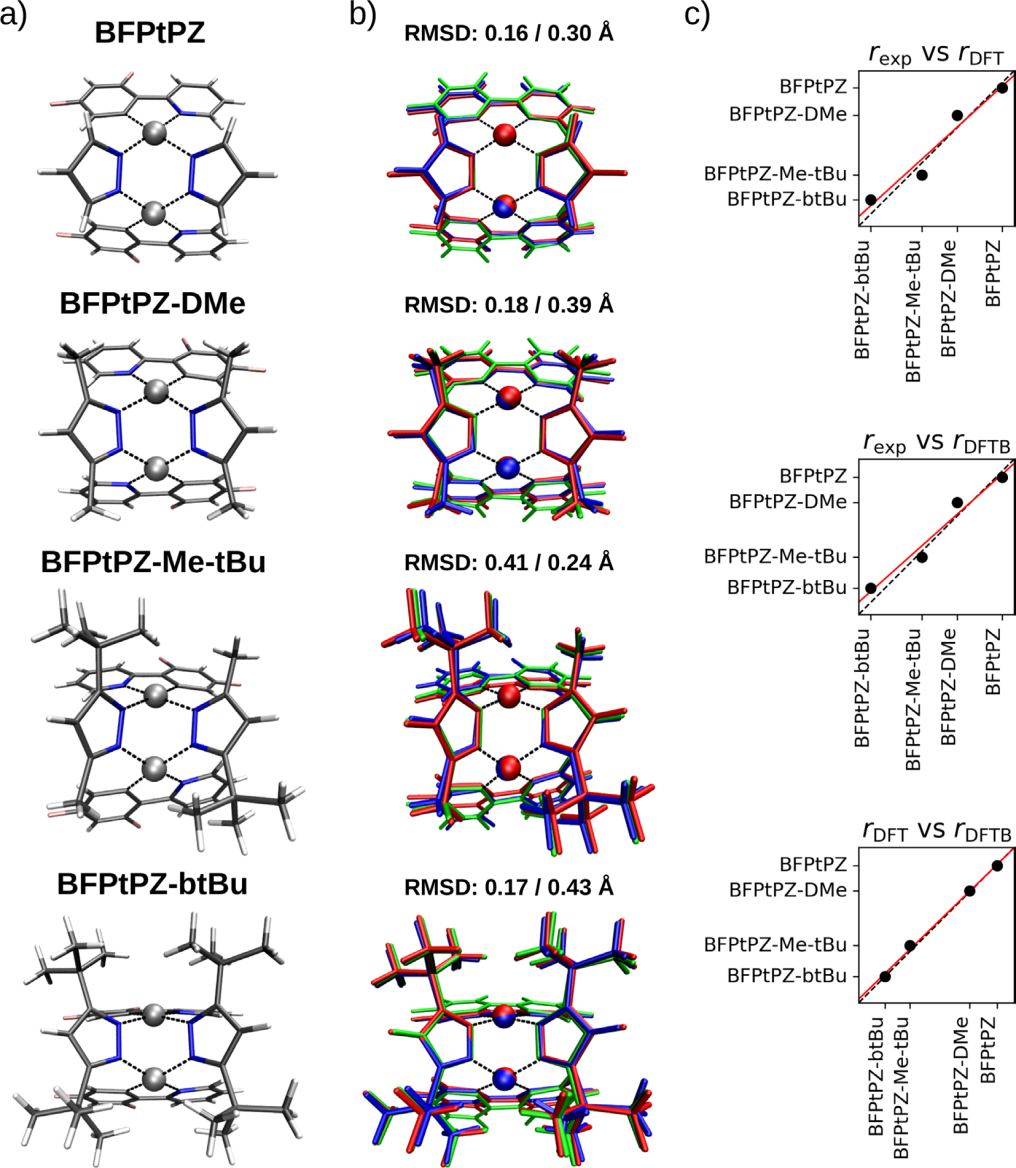
(a) Experimental structures of the differently substituted μ‐pyrazolate‐bridged cyclometalated platinum binuclear complexes (BFPtPZ) reported by Ma et al. [90]. (b) Overlay of the energy minimized structures obtained at B3LYP‐D3/triple‐zeta (red) and DFTB‐D3 (green) level of theory with the experimental reference (blue). The respective RMSD values have been determined for B3LYP‐D3 and DFTB‐D3 against the experimental structure for all non‐H atoms. (c) Correlation of the Pt‐Pt distances obtained at B3LYP+D3 and DFTB3 levels against the experimental reference values as well as against each other. The respective slopes, intercepts and correlation coefficients are listed in Table 5.

In order to provide an additional means of testing the quality of the parametrization, a highly challenging system was selected, namely the butterfly‐like platinum(II) binuclear pyrazolate complexes (BFPtPZ) reported by Ma et al. [90] (see Figure 4a). It was shown that depending on the substituents bound to the two pyrazolate ligands, the interatomic Pt⋯Pt distance can be steered over a comparably large margin. In this work, the dimethyl (DMe), methyl/*tert*‐butyl (Me‐tBU), bis‐*tert*‐butyl (btBU) variants along with the pristine BFPtPZ complex were considered, resulting in a variation of the Pt⋯Pt distance in the range of 2.83 to 3.38 Å. Thus, this set of complexes not only represents a challenging test case for the newly derived parameters but also enabled a detailed fine‐tuning of the Pt‐Pt repulsive potential, which could not be adequately validated in the other validation sets discussed above.

Each structure was subject to an energy minimization at B3LYP+D3/cc‐pVTZ and DFTB3 level of theory. An overlay of the respective minimum configurations with the experimental structure is shown in Figure 4b along with the corresponding RMSD values (excluding H‐atoms). While overall B3LYP+D3 tends to yield lower RMSD values, the structural description achieved using both methods shows a very good agreement with the crystal structure. Again, it should be noted that energy minimizations within a vacuum environment are expected to result in minor structural relaxations compared to configurations extracted from a bulk environment. Moreover, as already discussed above, deviations observed in the DFTB3 calculation are not solely the result of the newly derived parameters but are also influenced by the semi‐empirical nature of all interactions within the 3ob set (e.g., C–C, C–N, etc.).

**TABLE 5:** Slope, intercept and R value obtained from the correlation plot of the Pt·Pt distance in the BFPtPZ complexes shown in Figure 4c.

	Slope	Intercept/Å	R 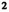
exp vs DFT	0.910	0.290	0.947
exp vs DFTB	0.892	0.349	0.962
DFT vs DFTB	0.972	0.092	0.997

In Figure 4c, the correlation between the Pt⋯Pt distances is depicted, the respective parameters for the linear fit are compared in Table 5. It can be seen that the DFT and DFTB methods yield highly similar deviations from the experimental structure, which can be attributed to the above‐mentioned difference between a gas‐phase and bulk crystal environment. This conclusion is highlighted when correlating the distances obtained from DFT and DFTB with each other, resulting in a near‐perfect correlation. In addition to a slope and R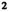 value close to 1, the associated intercept is notably reduced in the correlation between the theoretical methods.

These results demonstrate that the newly derived parameters are capable of describing this highly complex binuclear coordination complex with a high degree of accuracy compared to more demanding DFT calculation results and the experimental reference.

#### 3.3.3 Bond Dissociation Energies and Proton Affinities

To complement the structural benchmark, sequential bond dissociation energies and proton affinities were calculated for different small metal‐ligand complexes. Note that the energy of an isolated proton is not zero in DFTB due to energy contributions arising from the Hubbard parameter. In the original work on the DFTB3 method, the energy contribution associated with a single proton is outlined in detail, and the reported value of 151.04 kcal mol^−^^1^ obtained at PBE level of theory has been adopted in this work [43].

The deviations from reference methods (MP2) listed in Tables 6 and 7 are well within a reasonable tolerance with a mean deviation amounting to ≈6.7 kcal mol^−^^1^ on average. This is comparable to those obtained in the parametrization of Zr [7], Mg [54] Zn [54] and Cu [52] amounting to approx. 4, 4, 5 and 6 kcal mol^−^^1^, respectively. Consistent with their more intricate electronic structures, especially in the ionic state, copper and platinum display the largest deviations between DFTB predictions and quantum‐chemical reference data.

**TABLE 6:** Sequential ligand dissociation energies of molecules containing platinum: deviation from MP2/c‐cpVTZ, all energies are given in kcal mol^−^^1^.

Molecule	MP2	DFTB3/3ob + Pt
${\left[\text{Pt}{\left({\text{C}}_{2}{\text{H}}_{5}\text{N}\right)}_{3}\right]}^{2-}$	−88.4	−10.1
${\left[\text{Pt}{\left({\text{C}}_{2}{\text{H}}_{5}\text{N}\right)}_{4}\right]}^{2-}$	−89.7	−5.5
${\left[\text{Pt}{\left(\text{CO}\right)}_{2}\right]}^{2+}$	−81.7	−0.8
${\left[\text{Pt}{\left(\text{CO}\right)}_{3}\right]}^{2+}$	−106.8	6.3
${\left[\text{Pt}{\left(\text{CO}\right)}_{4}\right]}^{2+}$	−79.2	−11.7
${\text{PtH}}_{2}$	−402.5	10.2
${\left[\text{Pt}{\left({\text{H}}_{2}\text{O}\right)}_{4}\right]}^{2+}$	−74.1	−13.4
${\left[\text{Pt}{\left({\text{NH}}_{3}\right)}_{2}\right]}^{2+}$	−109.9	6.4
${\left[\text{Pt}{\left({\text{NH}}_{3}\right)}_{3}\right]}^{2+}$	−105.2	−8.7
${\left[\text{Pt}{\left({\text{NH}}_{3}\right)}_{4}\right]}^{2+}$	−83.8	−3.3
${\left[\text{Pt}{\left(\text{OH}\right)}_{4}\right]}^{2-}$	−7.9	−4.2
MAD		7.3
MAX		13.4

**TABLE 7:** Proton affinities of molecules containing platinum; deviation from MP2/cc‐pVTZ, all energies are given in kcal mol^−^^1^.

Molecule	MP2	DFTB3/3ob + Pt
${\left[\text{Pt}{\left({\text{H}}_{2}\text{O}\right)}_{2}\right]}^{2+}$	−102.3	−4.0
${\left[\text{Pt}{\left({\text{H}}_{2}\text{O}\right)}_{3}\right]}^{2+}$	−102.3	0.4
${\left[\text{Pt}{\left({\text{H}}_{2}\text{O}\right)}_{4}\right]}^{2+}$	−126.7	7.3
${\left[\text{Pt}{\left({\text{NH}}_{3}\right)}_{3}\right]}^{2+}$	−127.7	−15.9
${\left[\text{Pt}{\left({\text{NH}}_{3}\right)}_{4}\right]}^{2+}$	−164.1	−5.1
${\left[\text{Pt}{\left({\text{C}}_{3}{\text{H}}_{3}{\text{N}}_{2}\right)}_{3}\right]}^{2-}$	−199.1	−0.2
MAD		15.9
MAX		5.5

Note that due to the approximations inherent to the DFTB method and the complex properties of platinum, the treatment of systems with a small number of atoms and a net charge or charged ligands such as $\text{Pt}{\left({\text{CH}}_{3}\right)}_{2}$ and ${\left[\text{Pt}({\text{H}}_{2}\text{O})\right]}^{2+}$ tends to perform unsatisfactory in case of the calculations of sequential bond dissociation energies and proton affinities. However, if the treated platinum atom is well‐coordinated, for example, the investigation of fully coordinated ${\text{Pt}}^{\text{II}}$ complexes such as cisplatin or the QM/MM simulations of hydrated ${\text{Pt}}^{\text{II}}$ complexes, the performance of the parametrization is notably improved as shown by the associated simulation data discussed below.

#### 3.3.4 QM/MM MD Simulations of Pt(II) Complexes in Aqueous Solution

In order to further validate the performance of the new parameters beyond energy minimizations, hybrid QM/MM MD simulations of various ${\text{Pt}}^{\text{II}}$ complexes in aqueous solution were performed. The primary objective was to assess whether any dissociation or fragmentation of the complexes occurs in QM/MM MD simulations conducted at room temperature. All QM/MM MD simulations employed the QMCF approach [78, 79, 80], with a QM region comprising the first and second solvation shells of ${\text{Pt}}^{\text{II}}$ (corresponding to a QM‐region radius of 6.5 Å). This setup enables an assessment of whether the presence of water molecules treated at the DFTB3 level could induce fragmentation reactions arising from inadequate parametrization.

For the QM/MM MD simulations of ${\text{Pt}}^{2+}$ and ${\left[\text{Pt}{\left({\text{NH}}_{3}\right)}_{4}\right]}^{2+}$ in aqueous solution, the simulation setup and starting configurations were adopted from previous works [76, 77], with the newly developed DFTB3 parameters applied in the QM region. For cis‐platinum and ${\left[{\text{PtCl}}_{4}\right]}^{2-}$, the equilibrated structure of the ${\left[\text{Pt}{\left({\text{NH}}_{3}\right)}_{4}\right]}^{2+}$ simulation was used, and two or four NH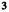 ligands were replaced by Cl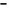, respectively. Following an equilibration period of 40 000 to 50 000 MD steps (20 to 25 ps), sampling was performed for an additional 200 000 steps (100 ps), except for ${\left[{\text{PtCl}}_{4}\right]}^{2-}$, where the structural motif of the complex was already well‐defined after 50 000 MD steps (25 ps).

RDFs derived from the simulation trajectories are shown in Figures S7–S10 of the Supporting Information. For the first two species, the RDFs are in good agreement with previous QM/MM MD studies [76, 77], considering the approximations inherent to the DFTB method. Both the shapes of the RDFs and the associated integration curves fall within a reasonable range, given the challenges associated with modeling metal‐organic structures in aqueous solution.

The complex solvation behavior of Pt(II) in aqueous solution has also been characterized previously [91] using Car‐Parrinello MD simulations [92] at the PBE level of theory [42]. This study reported loosely axial hydration and anionic solvation of axial ${\text{H}}_{2}\text{O}$ molecules, with a Pt‐H distance of approximately 2.55 Å. A similar motif was observed in the DFTB‐based QM/MM MD simulations, with a Pt‐H distance of 2.56 Å.

In summary, the QM/MM MD simulations provide clear evidence for the stability of the various ${\text{Pt}}^{\text{II}}$ complexes in aqueous solution, confirming the reliability of the newly developed Pt parameters to describe Pt‐containing systems at a nonzero temperature.

#### 3.3.5 DFTB MD Simulations of cisplatin@MOF Systems

In order to demonstrate that the extension of the 3ob parameter set to include the element Pt enables the study of complex systems, the chemotherapeutic drug cisplatinum (${\text{PtCl}}_{2}{\left({\text{NH}}_{3}\right)}_{2}$) has been embedded into the Zn‐ and Zr‐based MOF compounds ZIF‐8 and UiO‐66. Both materials have recently attracted attention as potentially promising vehicles for advanced drug‐delivery systems. For instance, in vitro and in vivo studies by Balsamy et al. [93] and Yang et al. [94] have reported anticancer and antibacterial properties of nanosized ZIF‐8 compounds in the presence of Fe, discussing potential release under pH conditions typically associated with tumor cells and applications within the context of tumor radiotherapy. Similarly, cisplatinum has been successfully embedded into UiO‐66‐type MOFs [27, 95].

**FIGURE 5:**
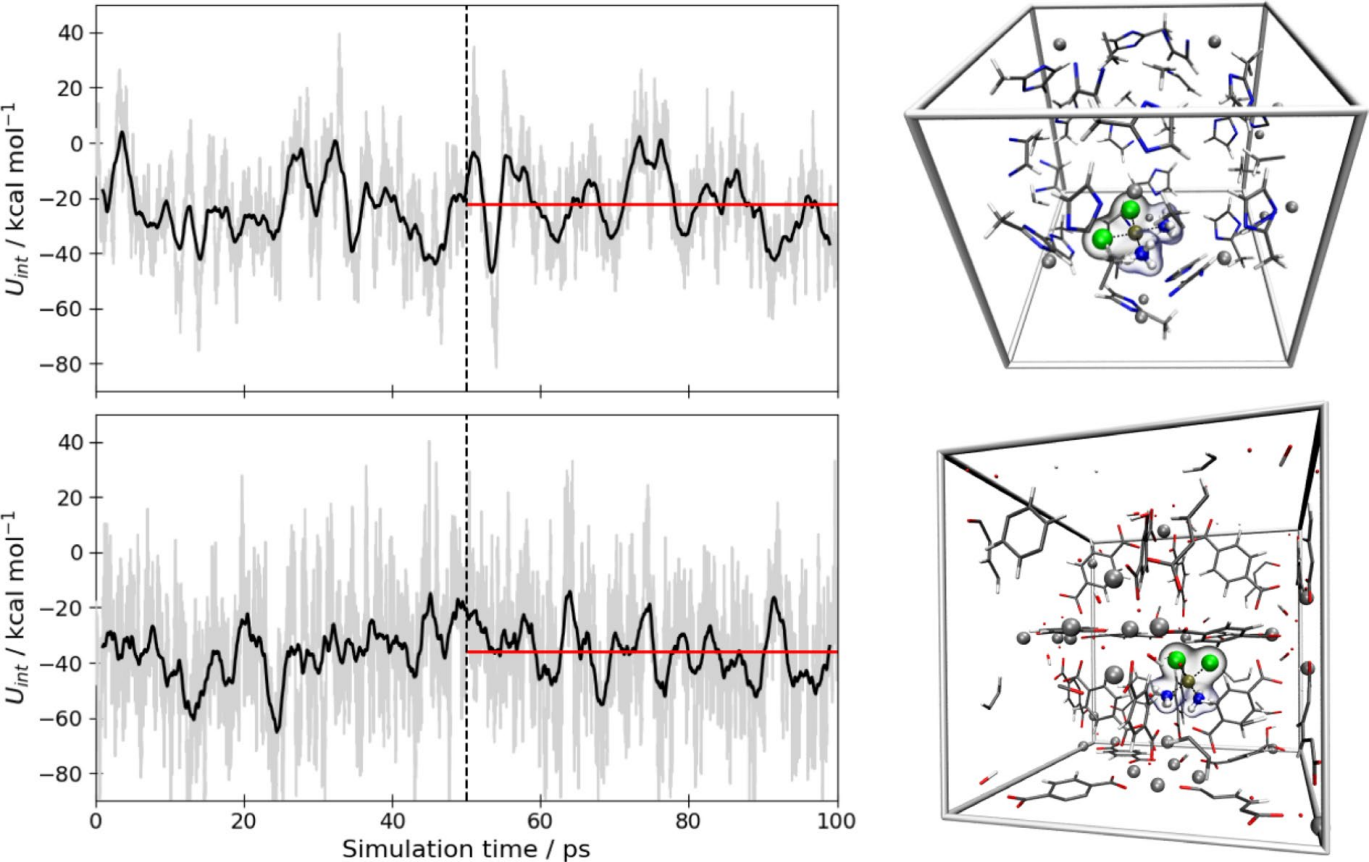
Left: Time evolution of the host‐guest interaction energy obtained via DFTB3 MD simulation of cisplatinum embedded in ZIF‐8 (top) and UiO‐66 (bottom). The black line indicates a moving average with a window size of 1 ps. The average values for $U_{\text{Int}}$ were determined considering the last 50 ps of the simulation. Right: Screenshots taken from the DFTB3 MD simulation depicting the typical interaction of the guest molecule with the ZIF‐8 (top) and UiO‐66 (bottom) host material.

The simulations were performed in analogy to previous successful MD studies of guest@MOF systems using DFTB3 in conjunction with the 3ob parameter set [7, 34, 35, 36, 37, 38, 39, 40]. In Figure 5 the time series of the interaction energy between the guest molecule and the MOF host along with the typical interaction motifs observed in the DFTB3 MD simulations are depicted.

Similar as in the previous studies the instantaneous interaction energies $U_{\text{Int}}$ were determined as

(9) $$U_{\text{Int}}=U_{\mathrm{guest@MOF}}-\langle U_{\text{guest}}\rangle -\langle U_{\text{MOF}}\rangle$$

with $U_{\mathrm{guest@MOF}}$ corresponding to the instantaneous internal energy obtained for the combined guest@host system at a given time step, $\left\langle U_{\text{guest}}\right\rangle$ and $\left\langle U_{\text{MOF}}\right\rangle$ are the average internal energy values determined in separate MD runs of the pristine MOF and the guest molecule at identical simulation settings. The average values for the interaction energy were determined as −22.2 and −35.9 kcal mol^−^^1^ in the case of cisplatin embedded in MOF‐5 and UiO‐66, respectively. While no experimental or theoretical reference data are available for direct comparison, the obtained interaction energies fall well within the typical range reported for polar molecular species embedded in various MOF hosts [36, 39, 96, 97].

Although the total sampling time of 100 ps is too short to fully capture the structural dynamics of the host‐guest interactions, the aim of these exemplary MD simulations was to demonstrate that the newly developed DFTB parameters for platinum enable stable simulations of these comparably complex systems under periodic boundary conditions.

It should be noted that empirically parameterized potential models (i.e., molecular force fields, FF) also provide an effective approach to model a broad range of guest@MOF systems [98, 99, 100] with the computational efficiency of FF approaches being a key advantage. However, the accuracy of such models can deteriorate in situations where strong polarization effects, significant charge transfer, or many‐body interactions play a decisive role, and several studies have highlighted these limitations in the context of MOF systems. While force field descriptions generally perform well to describe structural and mechanical properties of pristine MOF systems [99], they tend to struggle when applied to systems containing open metal sites [101] or when capturing the subtleties of guest‐host interactions within the porous network [102, 103, 104, 105]. In these cases DFTB‐based simulations are capable of bridging the gap between a pair‐wise additive force field description and highly demanding DFT approaches, enabling MD simulations up to nanosecond range.

## 4 Conclusion and Outlook

In this work, the creation of accurate DFTB parameters was outlined with the example of the highly challenging transition metal Pt. The outlined procedures are aimed at a straightforward parametrization of interactions between elements from the s‐, p‐, and d‐blocks of the periodic table. A detailed set of scripts for the parametrization procedure was provided to make the parametrization steps more accessible to the computational chemistry community.

Validation of the parameters involve (i) electronic band structures of bulk Pt, (ii) RMSD values and average bond distance deviations determined for more than 1300 Pt‐based compounds taken from the CCDC database and selected MP2 optimized structures, (iii) a detailed benchmarking of the challenging butterfly‐like platinum(II) binuclear pyrazolate complexes and (iv) selected sequential bond dissociation energies and proton affinities against MP2 reference data. To assess the performance of the developed parameters at nonzero thermal conditions, QM/MM MD simulations of different Pt‐containing complexes as well as DFTB MD simulations of cisplatinum embedded in the MOF host materials ZIF‐8 and UiO‐66 were provided.

These highly successful benchmarking result of this diverse validation set confirms a reliable extension of the 3ob DFTB parameter to include a vast array of Pt‐based compounds, thereby broadening the chemical space covered by the DFTB3 method to enable rapid and accurate investigations of platinum‐containing compounds.

## Funding

This work was supported by the Austrian Science Fund (Grant No. P 35427‐NBL).

## Conflicts of Interest

The authors declare no conflicts of interest.

## Supporting information




**Data S1**: Visualization of quadratic and Wood‐Saxon confinement potentials; Visualization of Pt‐{H, C, N, O, F, Cl, Na, Mg, Zn, Zr, Pt} repulsive potentials; Visualization of selected element‐element repulsive potentials in the 3ob parameter set; Visualization of selected geometries of the benchmark calculations; RDFs derived from the QM/MM MD simulation trajectories of Pt‐containing complexes in aqueous solution; Workflow summary for the construction of DFTB parameters; Exemplary python scripts for the construction of DFTB parameters; Listing of CCDC identifiers and corresponding RMSD values.

**Data S2**: Slater‐Koster files for the interactions of Pt‐{H, C, N, O, F, Cl, Na, Mg, Zn, Zr, Pt} and STO‐coefficients for Pt.

## Data Availability

The authors have nothing to report.
