# Supplementary material for: DFTB Parametrization at the Example of Platinum—Implementation, Validation and Practical Considerations
Source: J Comput Chem. 2026 Mar 11;47(7):e70342. doi: 10.1002/jcc.70342 (PMC12979722; doi:10.1002/jcc.70342)
Supplement: Supplementary file 1 — Data S1: Visualization of quadratic and Wood‐Saxon confinement potentials; Visualization of Pt‐{H, C, N, O, F, Cl, Na, Mg, Zn, Zr, Pt} repulsive potentials; Visualization of selected element‐element repulsive potentials in the 3ob parameter set; Visualization of selected geometries of the benchmark calculations; RDFs derived from the QM/MM MD simulation trajectories of Pt‐containing complexes in aqueous solution; Workflow summary for the construction of DFTB parameters; Exemplary python scripts for the construction of DFTB parameters; Listing of CCDC identifiers and corresponding RMSD values. [file JCC-47-0-s001.pdf]

DFTB Parametrisation at the Example of Platinum - Implementation, Validation and Practical Considerations

Supplementary Information

Felix R. S. Purtscher, Armin Penz, Josef M. Gallmetzer, Jakob Gamper, Stefan Seiwald and Thomas S. Hofer*

Institute of General, Inorganic and Theoretical Chemistry

Center for Chemistry and Biomedicine

University of Innsbruck, Innrain 80-82, A-6020 Innsbruck, Austria

Tel.: +43-512-507-57111

Fax: +43-512-507-57199

December 17, 2025

*Corresponding author: t.hofer@uibk.ac.at

S1 Confinement Potentials

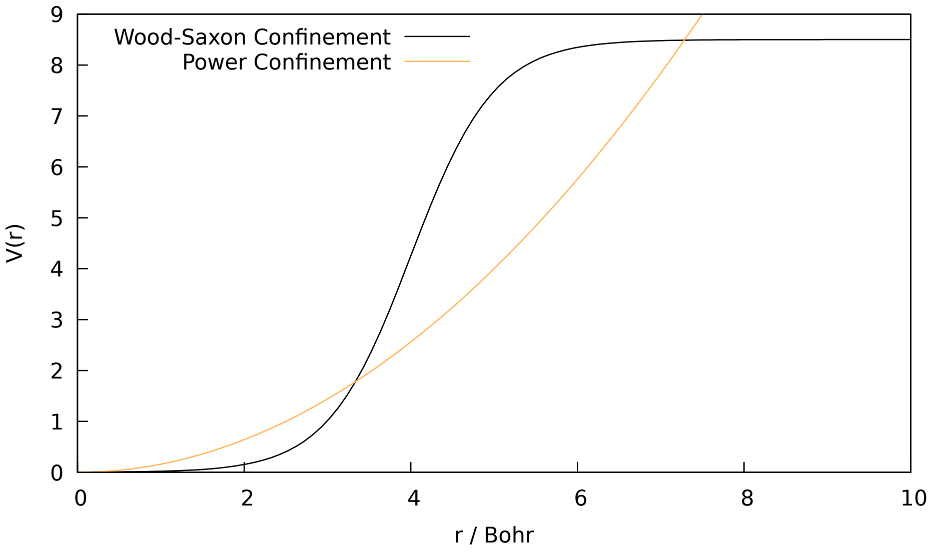

Figure S1: Depiction of the confinement potentials used in the creation of the Slater-Koster tables

S2 Platinum repulsive potentials

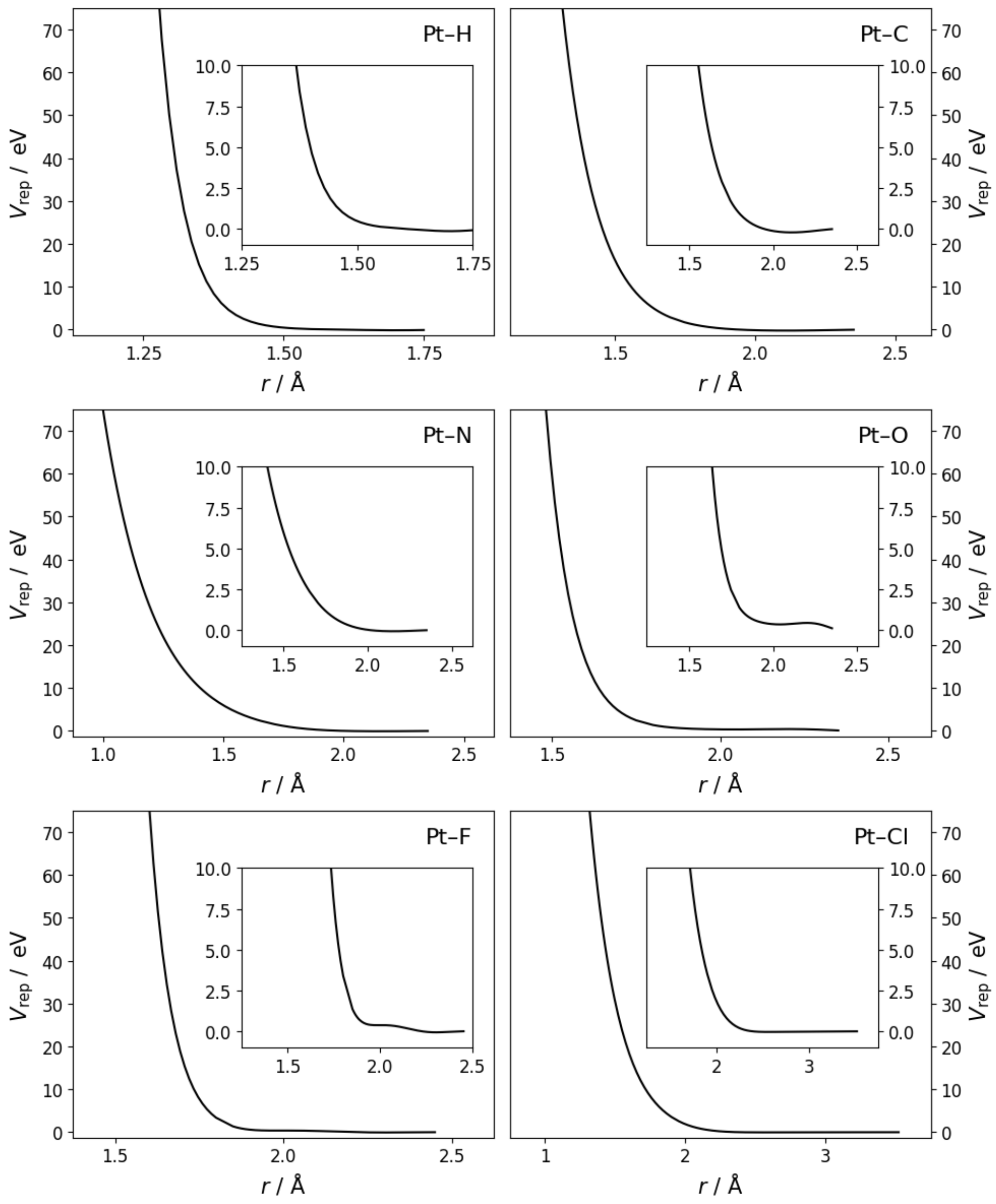

Figure S2: Depiction of the repulsive potentials of the respective atom pairs developed in this work. Shoulders and minima visible in the individual interactions are well within the expected range of typical repuslive potentials within the 3ob set (see Fig. S4 below).

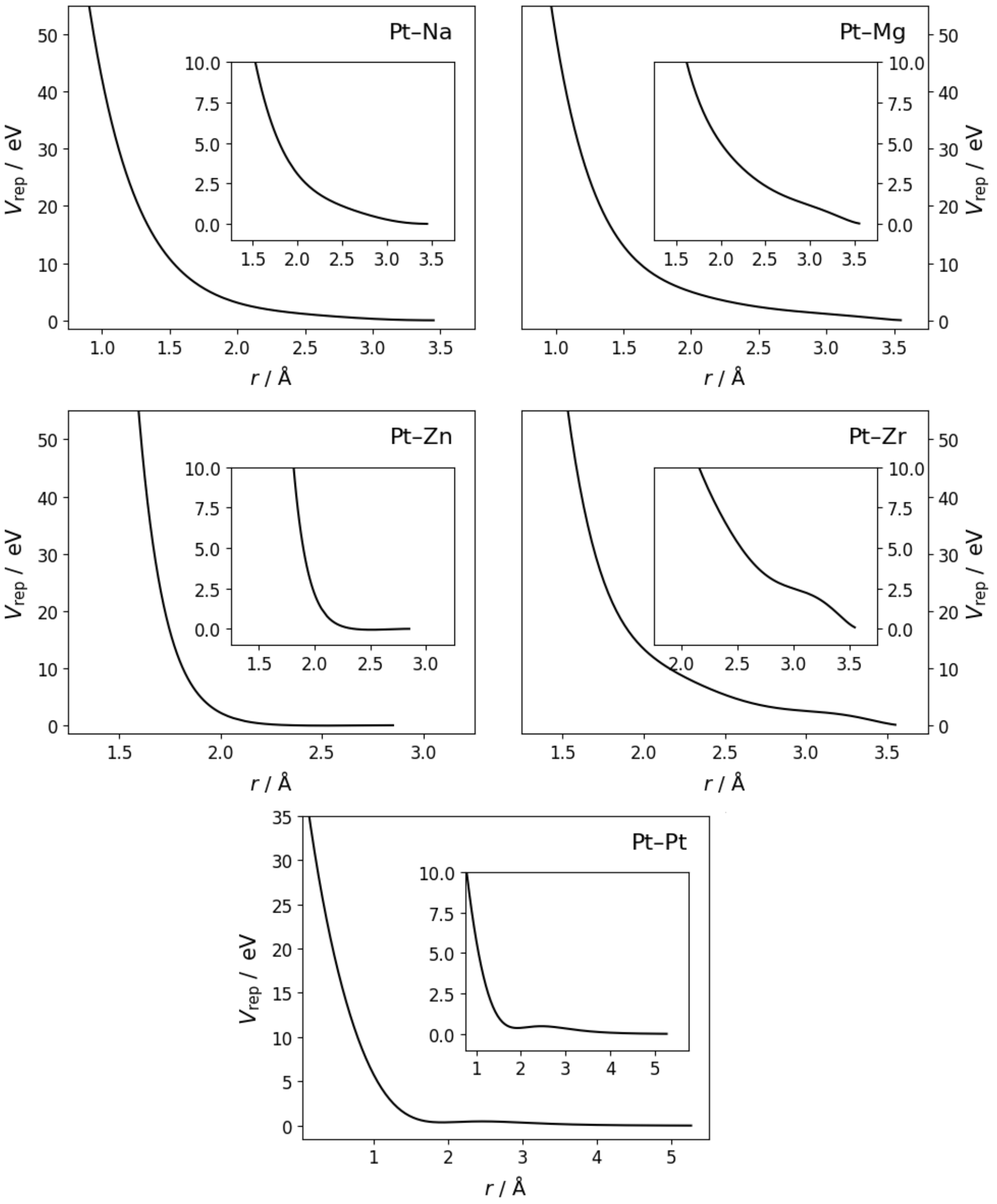

Figure S3: Depiction of the repulsive potentials of the respective atom pairs developed in this work. Shoulders and minima visible in the individual interactions are well within the expected range of typical repuslive potentials within the 3ob set (see Fig. S4 below).

S3 Comparison of 3ob repulsive potentials

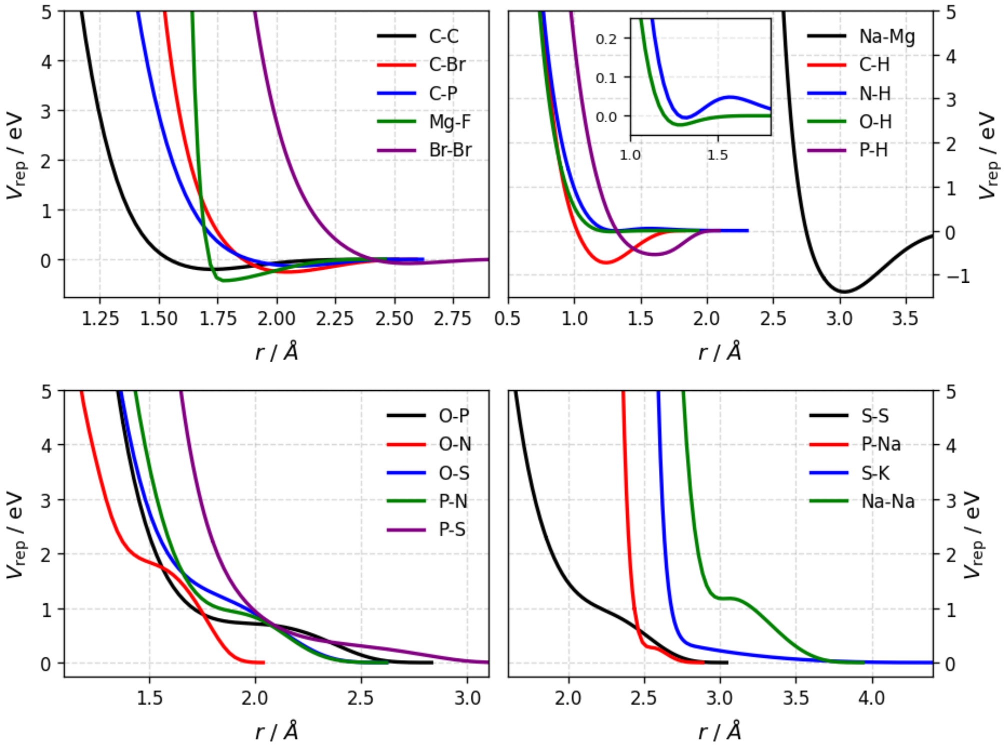

Figure S4: Depiction of the repulsive potentials of the respective atom pairs in the 3ob set.[1–4]

S4 Systems with largest deviations in the CCDC benchmark

cis-Dichloro-bis(cyclohexylamine)-platinum(II)
(RMSD: 0.857 Å; CCDC ID CCXAPT)

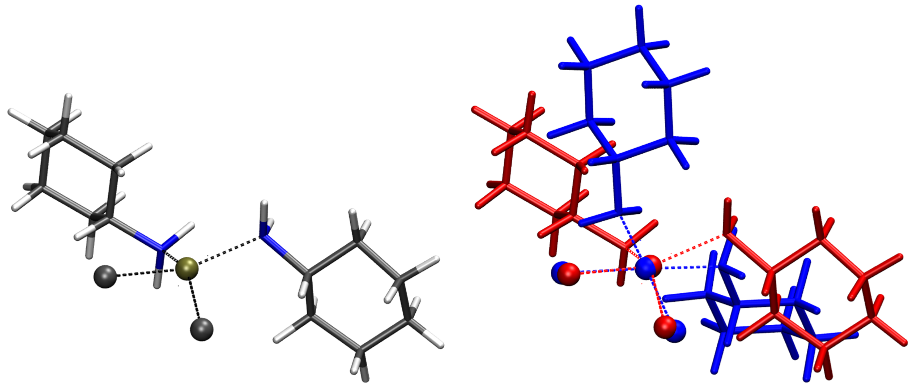

Dichloro-(N-(4-phenylbutyl)-1-(pyridin-2-yl) methanimine)-platinum(II)
(RMSD: 0.840 Å; CCDC ID: EKAFON)

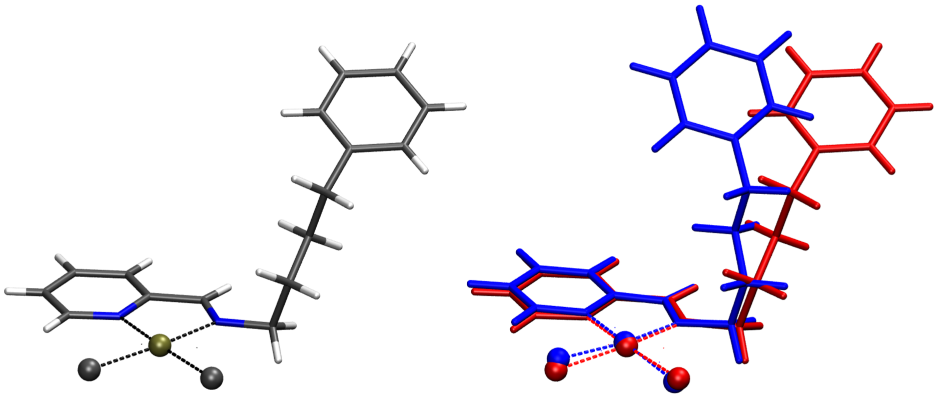

Figure S5: Structures showing the largest deviation between the crystal structure obtained from the CCDC database (left; red) and the DFTB calculations (blue). The high RMSD values are not the result of ill-defined DFTB parameters (as seen by the good agreement of the Pt coordination) but due to a relaxation of the comparably large and bulky ligands in the vacuum environment. It should be noted that the alignment of the ligands does not correspond to that obtained in the RMSD calculation but has been chosen to highlight the adequate description of the Pt-coordination.

S5 System with the largest deviation in the MP2 benchmark

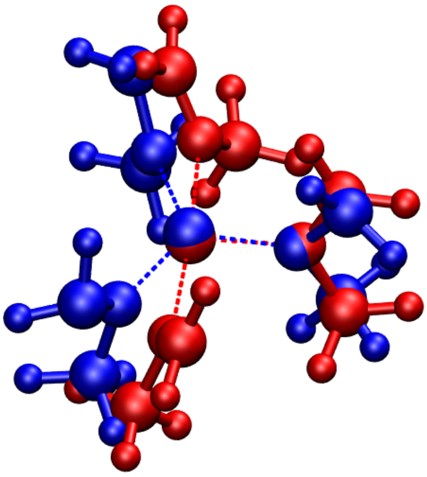

Figure S6: Superimposed geometries of the $[Pt(CH_3)_2N)_3]^-$ complex optimised at MP2/cc-pVTZ (red) and DFTB3/3ob (blue) level, respectivley. This systems represents the only significant outlier in the comparion between the MP2 and DFTB optimized structures resulting in an RMSD value of 0.59 Å. Due to the presence of an open coordination site, the weakly coordinated $(CH_3)_2N^-$ ligands in trans-position show a slight degree of relaxation associated also to a tilting indicating weak dispersive interactions between the associated CH_3 groups. It should be emphasized, however, that this deviation cannot be attributed exclusively to the newly derived Pt parameters, as contributions from other elements of the 3ob set (most prominently the N–C and C–C interactions) also play a significant role.

S6 Radial Distribution Functions obtained from QM/MM MD simulation data

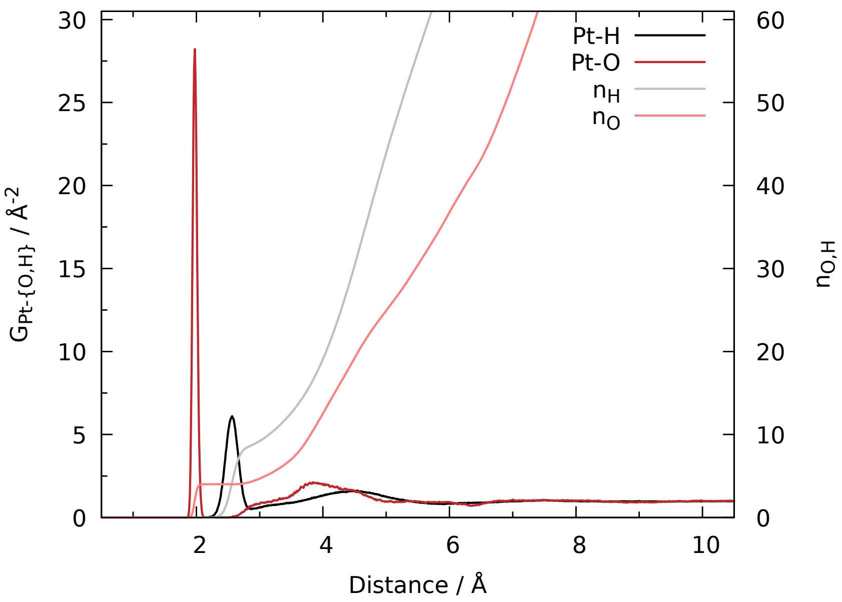

Figure S7: Radial distribution function obtained from of the QM/MM MD simulation of Pt^{2+} in aqueous solution.

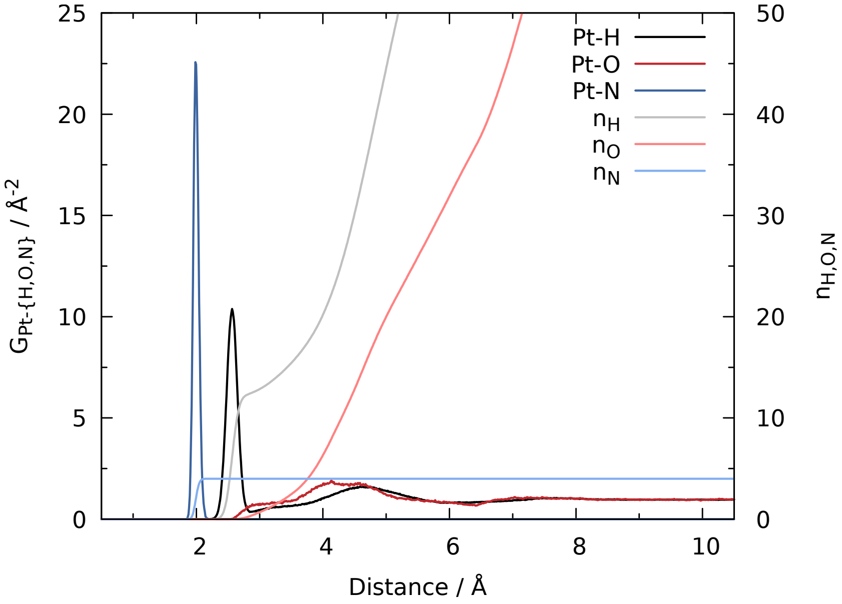

Figure S8: Radial distribution function obtained from the QM/MM MD simulation of $[Pt(NH_3)_4]^{2+}$ in aqueous solution.

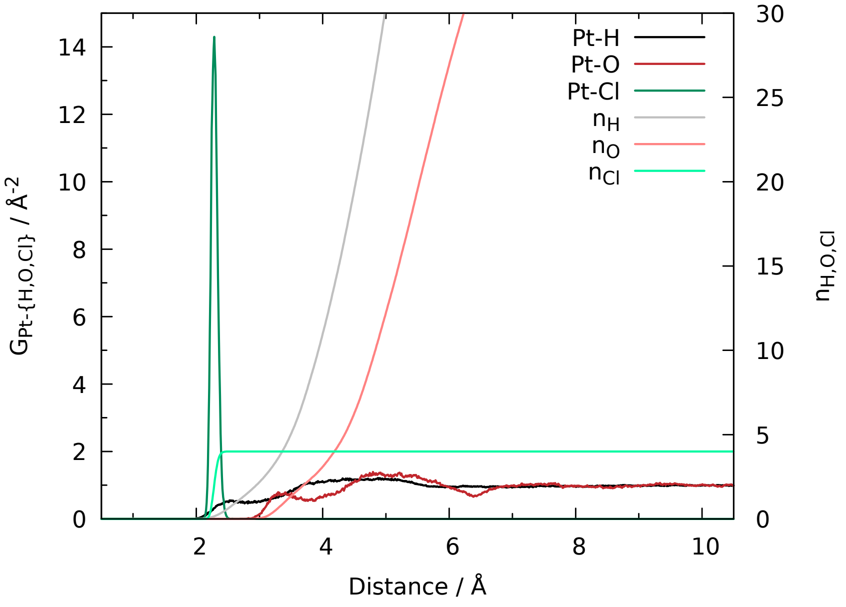

Figure S9: Radial distribution function obtained from the QM/MM MD simulation of $[PtCl_4]^{2-}$ in aqueous solution.

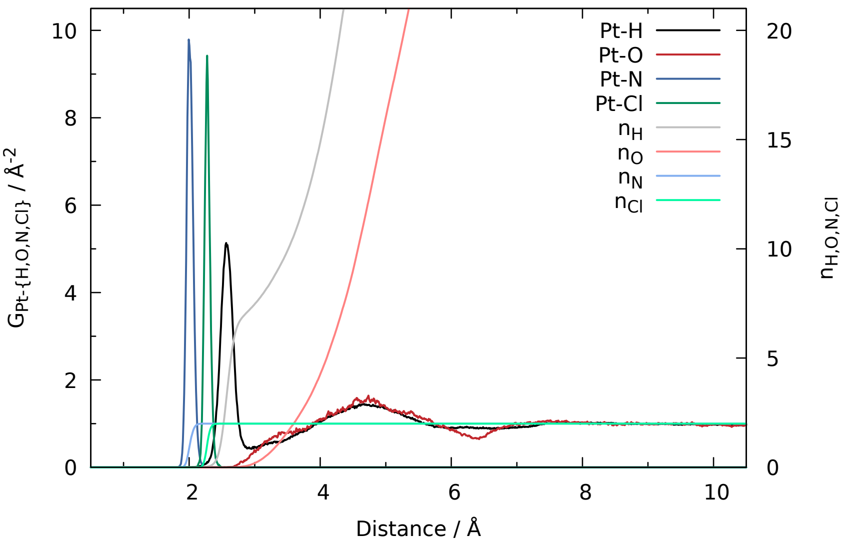

Figure S10: Radial distribution function obtained from the QM/MM MD simulation of cisplatin in aqueous solution.

S7 Workflow for the construction of DFTB Parameters

- Creation of the electronic part with *hotcent*[5, 6]
  - Calculate orbital eigenvalues
  - Calculate Hubbard values and derivatives
  - Adjust Hubbard values and derivatives (e.g. to correctly reproduce ionisation energies)
  - Calculate Slater-Koster tables, adjust confinement parameters for wave function and density confinement
- Creation of the repulsive part with *tango*[7] interfaced to the *atomic simulation environment*[8]
  - Select reasonable, preferably small reference systems
  - High-level calculations of reference systems, i.e. execute potential energy scans
  - Actual repulsive fitting - adjust r_{min}, r_{cut} values, check for inconsistencies in the repulsive potentials
- Benchmark, validation of the parameters
  - Structure optimisation and validation *via* RMSD calculations
  - Calculation of sequential bond dissociation energies
  - Calculation of proton affinities
  - QM/MM simluations of compounds in aqueous solution, calculation of RDFs

S8 Python Code

Listing 1: Python script for calculation of one center parameters of platinum, inlcuding the function for the determination of the hubbard derivatives

```python
from hotcent.atomic_dft import AtomicDFT
from hotcent.atomic_base import nl2tuple

element = 'Pt'
xc = 'GGA_X_PBE+GGA_C_PBE'


atom = AtomicDFT(element,
                 xc=xc,
                 convergence={'density':1e-8, 'energies':1e-8},
                 confinement=None,
                 configuration='[Xe] 4f14 5d9 6s1 6p0 ',
                 valence=['4f', '5d', '6s', '6p'],
                 scalarrel=True,
                 mix=0.15,
                 txt='-',
                 )

atom.run()
atom.info = {}

def get_hubbard_derivative(nl):
    configuration = atom.configuration.copy()
    n, l = nl2tuple(nl[:2])
    max_occup = 2 * (2*l + 1)
    occup = float(atom.configuration[nl])
    if occup == 0:
        scheme = [0.0, 0.01, 0.02]
    elif occup == max_occup:
        scheme = [max_occup, max_occup - 0.01, max_occup - 0.02]
    else:
        scheme = [occup - 0.01, occup, occup + 0.01]
    values = []
    for occ in scheme:
        atom.configuration[nl] = occ
        atom.run()
        values.append(atom.get_eigenvalue(nl))
    Ud = (values[0] + -2*values[1] + values[2])/(0.01**2)
    atom.configuration = configuration
    return Ud

atom.info['eigenvalues'] = {nl: atom.get_eigenvalue(nl) for nl in atom.valence}
atom.info['hubbardvalues'] = {nl: atom.get_hubbard_value(nl) for nl in atom.valence}
atom.info['hubbardderivs'] = {nl: get_hubbard_derivative(nl) for nl in atom.valence}

atom.info['spinconstants'] = {f'W{i[1]}{j[1]}': atom.get_spin_constant(i, j) for i in atom.
    valence for j in atom.valence}

print(atom.info)
```

Listing 2: Python script for the initial band fit of platinum

```
from ase.spacegroup import crystal
from ase.io.jsonio import read_json, write_json
from ase.data import atomic_numbers, covalent_radii
from hotcent.atomic_dft import AtomicDFT
from hotcent.confinement import PowerConfinement, WoodsSaxonConfinement
from hotcent.confinement_optimization import ConfinementOptimizer, DftbPlusBandStructure

rcov = covalent_radii[atomic_numbers['Pt']]

element = 'Pt'
xc = 'GGA_X_PBE+GGA_C_PBE'

conf = PowerConfinement(r0=6.9, s=2)

es = -0.20734
ep =  -0.03096
ed = -0.22595
ef = -2.576479470158313,

us = 0.2897
up = us
ud = 0.238

atom = AtomicDFT(element,
                 xc=xc,
                 convergence={'density':1e-8, 'energies':1e-8},
                 confinement=conf,
                 configuration='[Xe] 4f14 5d9 6s1 6p0',
                 valence=['5d', '6s', '6p'],
                 scalarrel=True,
                 mix=0.15,
                 txt=None,
                 )
atom.info = {}
atom.info['eigenvalues'] = {'6s': es, '6p': ep, '5d': ed}

atom.info['hubbardvalues'] = {'6s': us, '6p': up, '5d': ud}
atom.info['occupations'] = {'6s': 1, '6p': 0, '5d': 9}


# Creating a DFTB+ band structure evaluator and
# supplying it with a reference (DFT) band structure
dpbs = DftbPlusBandStructure(Hamiltonian_SCC='Yes',
                             Hamiltonian_SCCTolerance=1.0e-6,
                             Hamiltonian_Dispersion_='DftD3',
                             Hamiltonian_Dispersion_Damping_='BeckeJohnson',
                             Hamiltonian_Dispersion_Damping_a1=0.746,
                             Hamiltonian_Dispersion_Damping_a2=4.191,
                             Hamiltonian_Dispersion_s6=1.0,
                             Hamiltonian_Dispersion_s8=3.209,
                             Hamiltonian_Dispersion_cutoff = 94.868329805051374,
                             Hamiltonian_Dispersion_cutoffcn = 40.000000000000000,
                             Hamiltonian_MaxAngularMomentum_='',
                             Hamiltonian_MaxAngularMomentum_Pt='d',
                             Hamiltonian_PolynomialRepulsive='SetForAll {Yes}',
                             Hamiltonian_ThirdOrderFull='Yes',
                             Hamiltonian_hubbardderivs_='',
                             Hamiltonian_hubbardderivs_Pt=-0.070, # value by skprogs
)

bs_cry_pt = read_json('bs_cry_pt.json')

atoms_pt = crystal(['Pt'],
             basis=[(0.0,  0.0,  0.0)],
             spacegroup=1,  #### Crystal17 PBE OPT Values ####
             cellpar=[ 2.7765287581, 2.7765287581, 2.7765287581, 60.0, 60.0, 60.0]
             )

#dia
```

```
dpbs.add_reference_bandstructure(bs_cry_pt, atoms=atoms_pt, kpts_scf=(4, 4, 4),
                                 reference_level='fermi', nsemicore=0, weight=1.00,
                                 distribution={'type': 'Boltzmann', 'kBT': 2.5})

confopt = ConfinementOptimizer(atom, N=1149, rmin=0.02, dr=0.02, stride=1,
                               superposition='density', xc='GGA_C_PBE+GGA_X_PBE')

initial_guess = {'Pt_6s, Pt_6p': PowerConfinement(r0=3.13, s=2, adjustable=['r0']),
                 'Pt_5d':        PowerConfinement(r0=5.82, s=2, adjustable=['r0']),
                 'Pt_n':         PowerConfinement(r0=6.91, s=2, adjustable=['r0'])}


vconf = confopt.run(dpbs.get_residual, initial_guess=initial_guess, tol=1e-2,
                    method='COBYLA', options={'maxiter': 1, 'rhobeg': 0.1})


bs_dftb_pt = dpbs.calculate_bandstructure(bs_cry_pt)
bs_dftb_pt.plot(filename='bs_dftb_pt.png', emax=20, emin=-20)

bs_cry_pt.plot(filename='bs_cry_pt.png', emax=20, emin=-20)
write_json('bs_dftb_pt.json', bs_dftb_pt)

atom.write_hsd(filename='Pt_wfc.hsd')
print('Done!')
```

Listing 3: Python script for the Slater-Koster table creation at the example of the Pt-F interaction

```
from hotcent.atomic_dft import AtomicDFT
from hotcent.confinement import PowerConfinement
from hotcent.offsite_twocenter import Offsite2cTable

# Define PBE functional according to Libxc
# https://libxc.gitlab.io/
xc = 'GGA_X_PBE+GGA_C_PBE'

##### Fluorine #####
# Parameters taken from the 3ob set
# Kubillus et al. J. Chem. Theory Comput. 11, 2014, 332-342 DOI: 10.1021/ct5009137

element1 = 'F'

dens_conf1 = PowerConfinement(r0=5.0, s=2)

wf_conf1 = {
             '2s': PowerConfinement(r0=2.8, s=2),
             '2p': PowerConfinement(r0=2.8, s=2)
           }

# Atomic definition - recommended values according to hotcent
# https://gitlab.com/mvdb/hotcent
atom1 = AtomicDFT(element1,
                  xc=xc,
                  convergence={'density':1e-8, 'energies':1e-8},
                  confinement=dens_conf1,
                  wf_confinement=wf_conf1,
                  configuration='[He] 2s2 2p5',
                  valence=['2s', '2p'],
                  scalarrel=True,
                  mix=0.25,
                  txt='-',
                  )
atom1.run()
atom1.info = {}

# Parameters taken from the 3ob set according to F-F.skf
# taken from https://github.com/dftbparams/3ob/releases
atom1.info['eigenvalues']   = {'2s': -1.095847, '2p': -0.408705}
atom1.info['hubbardvalues'] = {'2s':  0.5584,   '2p':  0.5584}
atom1.info['occupations']   = {'2s':  2,        '2p':  5}


##### Platinum #####

# Parameters created in this work based on sktables to represent the band structure of Pt(fcc)

element2 = 'Pt'

dens_conf2 = PowerConfinement(r0=6.91, s=2.0)

wf_conf2 = {
             '5d': PowerConfinement(r0=5.824160, s=2.0),
             '6s': PowerConfinement(r0=3.133646, s=2.0),
             '6p': PowerConfinement(r0=3.133646, s=2.0)
           }

atom2 = AtomicDFT(element2,
                  xc=xc,
                  convergence={'density':1e-8, 'energies':1e-8},
                  confinement=dens_conf2,
                  wf_confinement=wf_conf2,
                  configuration='[Xe] 4f14 5d8 6s2 6p0',
                  valence=['5d', '6s', '6p'],
                  scalarrel=True,
                  mix=0.25,
```

```
                 txt=None,
                 )
atom2.run()
atom2.info = {}


atom2.info['eigenvalues']   = {'6s': -0.20734, '6p': -0.03096, '5d': -0.22595}
atom2.info['hubbardvalues'] = {'6s':  0.2897,  '6p':  0.2897,  '5d':  0.2380}
atom2.info['occupations']   = {'6s':  2,       '6p':  0,       '5d':  8}


# Compute Pt-F Slater-Koster integrals and write to files
# Pt-F.skf and F-Pt.skf
rmin, dr, N = 0.02, 0.02, 1050

off2c = Offsite2cTable(atom1, atom2)
off2c.run(rmin, dr, N, superposition='potential', xc=xc)
off2c.write()

rmin, dr, N = 0.02, 0.02, 1050
off2c = Offsite2cTable(atom2, atom1)
off2c.run(rmin, dr, N, superposition='potential', xc=xc)
off2c.write()
```

Listing 4: Python script for the database creation for repulsive fitting of the $[PtF_4]^{2-}$ complex

```
# Script to convert data from a potential energy scan into an ASE database
# at the example of the [PtF_4]^(2-) complex

import os
import numpy as np
from ase.io import read
from ase.db import connect
from ase.calculators.singlepoint import SinglePointCalculator

dbfile = 'PtF4_training_data.db'
assert not os.path.exists(dbfile), 'Please remove the existing training.db file!'

db = connect(dbfile)

# Read distances and energy in Angstroem and eV from a two column file PES_eV.dat
# Note: distances are only required for debugging purposes
#       tango requires configuration data and energies
distances = np.genfromtxt("PES_eV.dat", usecols=0)
energies  = np.genfromtxt("PES_eV.dat", usecols=1)

# Create empty force array for all atoms
# No force matching considered in this work
f = np.zeros((5, 3))

# Read all configurations of the potential energy scan
# from a single xyz-file
traj = read("PtF4_scan_geometries.xyz", ":")


# Add structures to database
# Note: Cell definition is note required in this case
#       but is kept in the program for example purposes.
#       If no cell is required, periodicity can be
#       deactivated setting pbc to [0, 0, 0]
count = 0
for r, e in zip(distances, energies):
    traj[count].cell=[500.0, 500.0, 500.0]
    traj[count].pbc=[0, 0, 0]
    atoms = traj[count]
    calc = SinglePointCalculator(atoms, energy=e, forces=f)
    atoms.set_calculator(calc)
    db.write(atoms, relaxed=1, gaid=0, total_charge=-2)
    count += 1
    print(count)
```

Listing 5: Python script for the repulsive fitting of the Pt-F interactions

```python
from ase.data import atomic_numbers, covalent_radii
from tango.calculators import DftbPlusCalculator
from tango import TANGO

elements = ['Pt', 'H', 'O', 'N', 'F'] # List all elements in the scan
mam = {'Pt': 2, 'H': 0, 'O': 1, 'N': 1, 'F': 1}  # the maximum angular momenta for each element

mode = 'exp_poly'          # the functional form (exponential decay at short distances, then a polynomial)
fit_constant = 'element'   # allow for one constant energy shift for each element
kBT = 20.0                 # energy which defines the Boltzmann weights from the (relative) cohesive energies
kptdensity = 1.0           # the k-point density in points per Ang^-1
force_scaling = 0.00       # how the forces should be weighted w.r.t. the forces (no force matching in this work)
weight_distribution = 0.0 # relevant when the fitting structure have different stoichiometries (all structures considerd with same weight)

dbfiles = ['database/ptf2.db',
           'database/ptf4.db',
           'database/pth2f.db',
           'database/pth2o3f.db',
           'database/h3f.db',
           'database/hf6.db',
           'database/nh33f.db',
           'database/ox4_f4.db']

rcovPt   = covalent_radii[atomic_numbers['Pt']]
rcovPtH  = (covalent_radii[atomic_numbers['Pt']] + covalent_radii[atomic_numbers['H']])/2
rcovPtN  = (covalent_radii[atomic_numbers['Pt']] + covalent_radii[atomic_numbers['N']])/2
rcovPtO  = (covalent_radii[atomic_numbers['Pt']] + covalent_radii[atomic_numbers['O']])/2
rcovPtF  = (covalent_radii[atomic_numbers['Pt']] + covalent_radii[atomic_numbers['F']])/2

rcuts = {
          'Pt-H':  None,
          'Pt-N':  None,
          'Pt-O':  None,
          'Pt-F':  1.5 * 2 * rcovPtF,
          'H-H': None, 'H-O': None, 'H-F': None, 'H-N': None,
                       'O-O': None, 'O-F': None, 'O-N': None,
                                    'F-F': None, 'F-N': None,
                                                 'N-N': None
        }

rmins  = {'Pt-F':  0.8*2*rcovPtF}
powers = {'Pt-F':  range(2, 7)}

calc = TANGO(elements,
             DftbPlusCalc=DftbPlusCalculator,
             kptdensity=kptdensity,
             rmins=rmins,
             rcuts=rcuts,
             powers=powers,
             update_rcuts=False,
             fit_constant=fit_constant,
             kBT=kBT,
             mode=mode,
             force_scaling=force_scaling,
             maximum_angular_momenta=mam,
             weight_distribution=weight_distribution,
             norep_suffix="no_repulsion",
             max_procs = 3
             )

residual = calc.fit_repulsion(dbfiles, run_checks=False)

print('Residual:', residual)
```

Listing 6: Python script for obtaining platinum complex structures

```
import ccdc
from ccdc.search import SubstructureSearch
from ase.db import connect
from ase import Atoms

dbfile = 'pt_ccdc.db'
db = connect(dbfile)

subst_search = SubstructureSearch()
pt_subs = ccdc.search.SMARTSSubstructure("[Pt]")
subst_search.add_substructure(pt_subs)

hits = subst_search.search()
print(len(hits))

myhits = []

def ccdc_to_ase(mol):
    symbols = []
    coords = []

    for at in mol.molecule.atoms:
        if at.coordinates is not None:
            if (at.atomic_symbol == 'D'):
                at.atomic_symbol = 'H'
            symbols.append(at.atomic_symbol)
            coords.append((at.coordinates.x,  at.coordinates.y,  at.coordinates.z))
    atoms = Atoms(symbols, coords)
    print(atoms)
    return atoms

for hit in hits:
    if (len(hit.molecule.atoms) < 75):
        charge = hit.molecule.formal_charge
        ase_atoms = ccdc_to_ase(hit)
        db.write(ase_atoms, relaxed=1, gaid=0, total_charge=charge, identifier=hit.identifier)
```

Listing 7: Python script for filtering platinum complex structures to the element set for the parametrisations

```
from ase.db import connect
from ase import Atoms, formula
from ase.utils import formula_metal

all_elements = {'H', 'He', 'Li', 'Be', 'B', 'C', 'N', 'O', 'F', 'Ne',
                'Na', 'Mg', 'Al', 'Si', 'P', 'S', 'Cl', 'Ar',
                'K', 'Ca', 'Sc', 'Ti', 'V', 'Cr', 'Mn', 'Fe', 'Co', 'Ni',
                'Cu', 'Zn', 'Ga', 'Ge', 'As', 'Se', 'Br', 'Kr',
                'Rb', 'Sr', 'Y', 'Zr', 'Nb', 'Mo', 'Tc', 'Ru', 'Rh', 'Pd',
                'Ag', 'Cd', 'In', 'Sn', 'Sb', 'Te', 'I', 'Xe',
                'Cs', 'Ba', 'La', 'Ce', 'Pr', 'Nd', 'Pm', 'Sm', 'Eu', 'Gd',
                'Tb', 'Dy', 'Ho', 'Er', 'Tm', 'Yb', 'Lu', 'Hf', 'Ta', 'W',
                'Re', 'Os', 'Ir', 'Pt', 'Au', 'Hg', 'Tl', 'Pb', 'Bi', 'Po',
                'As', 'Rn', 'Fr', 'Ra', 'Ac', 'Th', 'Pa', 'U'}


my_3ob_elements = {'C', 'Cl', 'H', 'N', 'O', 'F', 'Pt'}

print(my_3ob_elements)

mols = []

dbfile = 'pt_ccdc.db'
db = connect(dbfile)

db_filtered_file = 'pt_ccdc_filtered_unique_ptchnozncl.db'
db_filtered = connect(db_filtered_file)

formula_prev = ''

for row in db.select():
    if  row.formula != formula_prev:
        mol = row.toatoms()
        myset = set( mol.get_chemical_symbols() )
        if myset.issubset(my_3ob_elements):
            db_filtered.write(mol, identifier=row.identifier)
    formula_prev = row.formula
```

Listing 8: CCDC identifiers of the validation set and RMSD values in Å

ABIWUD	0.457772	ACASEE	0.746943	ACATUT	0.301465	ACEQAA	0.086923
ACIHID	0.471574	AFABUG	0.156459	AFAHUN	0.462077	AFAJAV	0.061887
AFAPEC	0.574958	AFAPOM	0.201861	AGICIC	0.133000	AHEHIE	0.133008
AHOWIF	0.086627	AHUNIA	0.122935	AHUNUM	0.222071	AJELUZ	0.119477
AJEMAG	0.159156	AJESEN	0.279280	AJEXES	0.035875	AJIYEY	0.074704
AJUDAN	0.121459	AKOFEM	0.108801	AKUFIV	0.452370	AKUVUA	0.042879
ALAJON	0.378865	ALEDIE	0.622715	AMCBPT01	0.206520	ANITIB	0.330596
ANITOH	0.267540	APEBAB	0.189335	APEBIJ	0.329010	APEBOP	0.155249
APUNEF	0.352917	AQEGOS	0.135302	AQIFOY	0.243435	AQIHOA	0.179882
AQIKUI	0.235177	AQILAP	0.344987	AREHEK	0.242056	ASUPEK	0.078049
ASUPIO	0.271263	ATALAK	0.173987	ATHLPT	0.373844	AWOPOR	0.400795
AXUNIQ	0.285996	AXUTAO	0.137821	AYUKEN	0.398394	AYUWOJ	0.154517
AZOFEB	0.153882	BACZOW	0.194218	BADBIT	0.729070	BADXUB	0.474139
BADYEM	0.329453	BADYIQ	0.320999	BADYUC	0.295469	BAGLAW10	0.193169
BAKSUD	0.063895	BAMMUY	0.163129	BAMNAF	0.214320	BEPFUX	0.149208
BEQSEW	0.089687	BERDAE	0.234467	BESGUC	0.112554	BEVFIU	0.163076
BEYGIZ	0.149614	BIBFUN	0.098075	BIBGEY	0.387505	BIKSAQ	0.418700
BIVXIN	0.503993	BOBJOR	0.250795	BOFTEV11	0.126030	BOJQUO	0.238583
BOKGIR02	0.117087	BOLPOI	0.163123	BOSJAX	0.059332	BPYCPT	0.064588
BQDXPT01	0.038364	BUFQEA	0.344390	BUPJED	0.104051	BUPJON	0.149500
BUZZAY	0.351174	CABMAV	0.162683	CACDIT	0.463705	CAGDUJ	0.154850
CAHBOC	0.284818	CARTOE	0.239192	CARTUK	0.299048	CAVYAA	0.094765
CAVYEE	0.094107	CAYHOA	0.175743	CAZVEG	0.297074	CCAPGC11	0.229812
CCENPT	0.133011	CCLMIP	0.094478	CCLUPT	0.629328	CCPYPT	0.242168
CCXAPT	0.856746	CECRUX	0.148540	CEFJAY10	0.061166	CEFZEW	0.175374
CEJKUY	0.097483	CEKCAY	0.189207	CEKCIG	0.153048	CETEPT01	0.120973
CETEPT	0.186943	CEVGIU	0.096319	CHXNPT	0.035520	CIJZAY	0.160439
CILFEI	0.051481	CILVUO	0.343723	CIMVEB	0.087741	CIRREA	0.132520
CLMEPT	0.178554	CLPYPT	0.104284	CMADPT	0.140622	CMAPPT	0.079912
CNPLPT	0.390704	COFSOI	0.093373	COFTAV	0.587937	COFYED	0.451153
COMPUS	0.305114	COMQED	0.265487	COQGUL	0.743895	COQHAS	0.182077
COQHEW	0.213421	COQHOG	0.214211	CORNOM	0.415738	COSYOB	0.144022
COSYUH	0.411118	COTWIS	0.095128	COYDOK	0.085055	CPEAMP	0.209711
CPMOPT10	0.145146	CUBLUH	0.080929	CUFKEV	0.419395	CUHKEV01	0.249098
CUKLEA	0.072294	CUKRAB02	0.080818	CULWUB	0.275022	CUMYER	0.111427
CURVOC	0.252444	CUXVAS	0.444616	CUXWOH	0.682341	DADPON	0.234658
DAFMEF	0.190026	DAGNUX	0.198766	DALWES	0.151535	DALXIX	0.108999
DAMHOR	0.130284	DANQAK	0.055259	DANVUJ	0.476994	DAXRUQ	0.417147
DEDGUR	0.416666	DEDHAY	0.123606	DEGZEW	0.309548	DEGZIA	0.067604
DEHYAU	0.267536	DEHYEY	0.075496	DEHZEY	0.306928	DEJBEA	0.118606
DELVIZ	0.331198	DEMCOR	0.055897	DEMPOD	0.123519	DEPBEF	0.050581
DEQMAO	0.153579	DEQMES	0.306840	DEWSAC	0.107819	DIBLOQ	0.218690
DICTOY	0.101610	DICTUE	0.089321	DIDNOT	0.213557	DIFKAE	0.129851
DIGCON	0.099285	DIGROD	0.601498	DILKAK	0.225027	DILKUE	0.304073
DILXOO	0.146747	DIPTCL01	0.109060	DIQBAG01	0.223613	DIVGEU	0.319722
DIVXOV	0.028253	DIWQAE	0.118751	DOCQAN	0.183735	DOCQER	0.142600
DOCQIV	0.149730	DOCQIV02	0.153748	DODSEW	0.087806	DOFTAW	0.298352
DOGZII	0.054237	DOMPOL	0.107530	DOMPUR	0.127891	DOMRAZ	0.452972
DOQTEK	0.205235	DORXOA	0.216413	DORYER	0.109154	DORYIV	0.381685
DOXPEL	0.216475	DPACPT10	0.446279	DUBZIL	0.125758	DUBZOR	0.202235
DUKPEF	0.129805	DUNGOK	0.234706	DUNGUQ	0.187608	DUPGAW	0.338562
DUSHII	0.128549	DUSHOO	0.272789	DUTGII	0.179304	ECEVEN	0.091651
EDUMOF	0.647007	EDUQOL	0.090221	EDUSUS	0.276425	EFOCAD	0.222771
EFUQOO	0.069643	EGAZUI	0.117019	EGEYOG	0.436949	EHAGEZ	0.366911
EHAGID	0.385171	EHAPIN	0.058765	EHICIJ	0.048406	EHISUL	0.129340
EHOKAQ	0.072974	EHUMAY	0.151414	EJITUN	0.114855	EJOCIQ	0.264535
EJODAJ	0.337030	EKAFON	0.840248	EKINEU	0.151634	EKIPOD	0.125461
ELANIO	0.775382	ELUREI	0.100118	EMAYEW	0.262259	EMAYIA	0.098755
ENAVIB	0.151363	ENEJIS	0.311021	ENEJOY	0.079506	ENESIB	0.121978
EPEBUX	0.111434	EQICIR	0.275305	ERAPUI	0.115365	ERUMIQ	0.142634
ESUWUN	0.168948	ETAFEL	0.266357	ETALOA	0.188545	ETAQEW	0.156894
ETAQIA	0.146378	ETARAT	0.217827	ETUNIT	0.584930	EVUKIQ	0.108606
EVUKOW	0.347438	EWAVOP	0.427911	EWAWEH	0.071738	EWAWIL	0.087772
EWUZUU	0.207708	EXEBIU	0.028094	EXOLUZ	0.232716	EXUBAA	0.087983
EYIQEJ	0.246916	EYIQOT	0.460739	EYIQUZ	0.413508	EYUSAS	0.141737
EZAXUA	0.335449	EZOKIP	0.127660	EZOSIX	0.177336	EZOSOD	0.152724
EZOTOE	0.101735	FADNUU	0.149760	FAGXAN	0.292640	FAGXIV	0.133862
FAJWOC	0.444166	FALRUI	0.143273	FAMRIW	0.438463	FAMROC	0.652079
FAPRIA	0.192971	FAPVIE	0.205983	FAPVOK	0.207254	FAQQUM	0.329592

FASBOQ	0.232955	FATJIW	0.250892	FATPAR	0.142508	FAWYEH	0.430087
FAYKUP	0.459791	FAYLAW	0.071625	FAZDOB	0.090976	FAZDUH	0.393560
FEJGAG	0.525056	FEKGUA	0.114368	FEKHUB	0.250870	FEKJEN	0.225966
FELNER	0.257064	FELNIV	0.265388	FELPER	0.413623	FENZEH	0.121630
FERMAS	0.188199	FERMEW	0.308235	FERMIA	0.462370	FERSUQ	0.172709
FEVPUR	0.190453	FEXRUV	0.492695	FEXSAC	0.223140	FEXXAI	0.142994
FEXXEM	0.131333	FEXZAM	0.622790	FEYJAW	0.089513	FEYXOW	0.499124
FEZWEQ	0.166248	FIBMIN	0.168629	FIFJUA	0.473245	FIQCOY	0.112093
FIWYAM	0.160984	FODNER	0.173937	FODNOB	0.159802	FOHJUJ	0.332275
FOHKEU	0.306502	FOSWOA	0.059391	FOTSOX	0.241154	FOWTUG	0.292572
FOZCAY	0.134379	FOZCAY10	0.110855	FOZCEC	0.200751	FUSHEH	0.098764
FUSTIY	0.181590	FUZCIN	0.719297	GACYUH	0.236779	GAMKUC	0.141586
GAPJOW	0.344600	GAPROF	0.284951	GAQGIO	0.230374	GEBVUE	0.204001
GECGIE	0.110544	GECGOK	0.146563	GECGUQ	0.145539	GECHAX	0.222110
GEFNIR	0.073201	GEFNOX	0.207132	GEFVAR	0.207162	GEHQAM	0.199072
GEHQOC	0.080301	GEHQUI	0.087831	GEJTOH	0.195966	GEJTUN	0.117648
GEJVAV	0.320499	GEKQOF	0.084131	GEMPOG	0.349511	GENLUG	0.168342
GEPSEA	0.084146	GETTAE	0.090958	GEWFIB	0.121580	GEWRAD	0.139276
GICKUY	0.049315	GIDKEL	0.223684	GISJOH	0.364055	GIYJON	0.359423
GOFTEC	0.139867	GOKZAH01	0.452616	GOQPEH	0.774824	GOYJEL	0.237796
GOYTAR	0.224692	GUDCEN	0.081417	GUZKER	0.128499	HACRAF	0.067771
HACTIS	0.570609	HACTUE	0.576672	HAFDAU	0.413956	HAHHIJ	0.034878
HALCIK	0.424331	HARKUI	0.104948	HAVPEA	0.131649	HAXXOV	0.149153
HAXZEN	0.168372	HAZLEA	0.141668	HEFXIA	0.246427	HEHTAP	0.126652
HEKBIM	0.135297	HEKCAF	0.465161	HEKJAI	0.275048	HEKJEM	0.142695
HEQVOP	0.509603	HETTUW	0.087899	HEWNUS	0.207768	HEWPAA	0.455549
HEYYEQ	0.103740	HEYYOA	0.246946	HIGBUV	0.088969	HIGVUO01	0.196495
HIGVUO	0.127774	HIGVUO02	0.284645	HIJROI	0.250799	HILLUL	0.227199
HIPXEK	0.122993	HIQRAB	0.230789	HISNOM	0.426551	HIWHIH	0.151534
HIZCEB	0.413607	HOCSOH	0.041088	HOJRII	0.070175	HOJROO	0.568596
HOKTEH	0.181195	HORCOJ	0.141551	HORCUP	0.151404	HOVRUF	0.261483
HOWVEV	0.095596	HOWVIZ	0.192174	HOWVUL	0.237156	HOWWAS	0.188572
HOWWEW	0.135090	HUJNAC	0.101189	HUJTOY	0.184281	HUQJIM	0.082247
HUQJOS	0.077433	HUQJUY	0.126560	HUQKAF	0.119638	HURRAO	0.290145
HURRES	0.139502	HUSXEA01	0.076443	HUSXEA	0.155826	HUTDEG	0.524523
HUVRIC	0.337023	HUVROI	0.437316	HUXXON	0.149774	IBENIQ	0.116942
ICUVAD	0.140326	ICUVIL	0.209789	IDAREL	0.366971	IDUCEP	0.266472
IDUCIT	0.390659	IFAGEC	0.234712	IFEJIM	0.148655	IFIRIB	0.325913
IGEXEZ	0.155486	IGEXID	0.184192	IGILUH	0.107863	IHEBED	0.119460
IJISIF	0.051428	IJISOL	0.183277	IKUVIV	0.181017	IPEKEW	0.056116
IPEKOG	0.150730	IPIHAQ	0.083535	IPIHEU	0.144566	IQIROQ	0.141848
IQOHAX	0.532191	IQOHEB	0.544410	IREDIU	0.175541	IROZOE	0.219109
ISAWUV	0.525737	ISIWUF	0.060958	ISIXEQ	0.065760	ISNBZP	0.626769
IXIBOG	0.314553	IXUHOZ	0.211954	IYUDIQ	0.086706	IYUTOO	0.091631
IZOCAD	0.172914	IZUDUC	0.365430	JAFNOW	0.205394	JAHWUL	0.186341
JAJDAA	0.231022	JAMJIQ	0.406579	JAMLOY	0.044993	JAQMIA	0.335735
JARCAG	0.129363	JARSEA	0.159538	JARSEA10	0.159520	JAXHIC	0.039355
JAXQOP01	0.126541	JAXQOP	0.102362	JAZWUF	0.346445	JEBZOJ	0.073774
JEHPOB	0.111116	JEMXUY01	0.118917	JEMXUY	0.119880	JEMXUY02	0.119321
JEMXUY03	0.116840	JEMXUY04	0.094879	JEMXUY05	0.117127	JESGAP	0.171199
JEVHOH	0.084229	JEXWAN	0.687968	JEXXEQ	0.070094	JEXZES	0.298256
JILPEZ	0.164673	JIMCAK	0.108465	JISPOR	0.235603	JISPUX	0.156135
JOBPEX	0.060458	JOBPIB	0.056628	JOBPOH	0.314734	JOBPUN	0.069835
JOCYOQ	0.056016	JOCYUW	0.062880	JODBIO	0.351056	JODTEC	0.057877
JOKHOI	0.173336	JOQXAR	0.378988	JOSDAW01	0.334110	JOWBUV	0.070419
JOWCEG	0.091267	JOWCOQ	0.105378	JOWCUW	0.083257	JOWDAD	0.103370
JOWDEH	0.096509	JUCMAV	0.369132	JUMJUW	0.302715	JUSVUQ	0.484119
JUXBUC	0.172644	JUXCAJ	0.090991	JUXCOX	0.158581	JUZPUS	0.256023
KACDEZ	0.154552	KACDID	0.137046	KAJMIT	0.518591	KAKHOX	0.217343
KAKJAL	0.130822	KAKJEP	0.220825	KAKJIT	0.308149	KAKJUF	0.167160
KALZEF	0.204219	KALZIJ	1.079879	KAPQAX	0.126772	KAQHIW	0.229786
KARQOJ	0.215355	KARQUP	0.213351	KARROK	0.203951	KASPEB	0.318319
KATSOO	0.082283	KEFKIQ	0.416702	KEFMEN	0.176497	KEJGUE	0.426544
KEJHAL	0.147950	KEJNUK	0.066474	KELFEN	0.099792	KELLIY	0.197810
KEMJES	0.267718	KEPSUV	0.205193	KEQSOO	0.091850	KEQSUV	0.072812
KEXHOL	0.279570	KIDKOY	0.245210	KIGBIP	0.087181	KIGCIQ	0.064405
KIGCOW	0.085503	KIGDAJ	0.104649	KIGGEO	0.069042	KIGGIS	0.194053
KIPVAG	0.071087	KIPVEK	0.116396	KIQQUZ	0.147000	KIRQIL	0.303970
KIVKEH	0.233430	KIVKIL	0.557319	KIVKOR	0.330999	KIVKUX	0.457413
KIVLEI	0.679229	KIZZUO	0.163758	KODBIP	0.123440	KOFDUE02	0.147897

KOFDUE	0.272064	KOFMEA	0.605981	KOGWAG	0.384324	KOGWEK	0.070210
KOHBIU	0.045528	KOHBOA	0.305512	KOHTEH	0.251828	KOKTIP	0.282764
KOMKEC	0.607146	KONBEW	0.168780	KOXFEK	0.172217	KOZLUJ	0.172643
KUFNIJ	0.189777	KUFNOP	0.151735	KUFNUV	0.112756	KULHEF	0.231352
KURQOE	0.056322	KUSNIV	0.486069	LACQOW	0.146776	LACQUC	0.081647
LAJBOO	0.505747	LAMFAG	0.434344	LAMMOB	0.247260	LAZRUD	0.234179
LECRAN	0.304356	LEDLOW	0.393833	LEDMAJ	0.370695	LEJROI	0.248477
LEJXUU	0.157126	LEVBAT	0.177865	LEWQOU	0.238952	LEWXAN	0.293249
LEYKUV	0.169027	LICMIU	0.355015	LIHPIB	0.466829	LIHYOQ	0.067499
LIMKAV	0.554577	LIMLEA	0.556435	LIMRAB	0.226176	LINLEC	0.080614
LIPFIZ01	0.385347	LIWNOV	0.406947	LIXHIJ	0.130461	LIXTER	0.379431
LIXTOB	0.108783	LODBUB	0.441935	LOGRUV	0.084933	LOGSAC	0.079358
LOJPEG	0.503092	LOLSEL	0.561248	LONWER	0.436848	LOQTUH	0.371091
LOQVAP	0.082749	LOQVUJ	0.490994	LOTNAJ	0.085953	LUDROT	0.079789
LUFPAF	0.307802	LUHXUK	0.058338	LUKLEK	0.322872	LUKYOF	0.115366
LULPAK	0.109710	LUZBOZ	0.127900	MAHZOM	0.259649	MAJBOR	0.273003
MALWAA	0.164947	MALWOO	0.118244	MALWUS	0.536446	MALXAZ	0.094223
MAMGUE	0.236222	MANROJ	0.269864	MAQBUD	0.355996	MASDER	0.175474
MAWXUD	0.556852	MEBSER	0.321466	MEHYUU	0.076897	MEHZEF	0.150242
MEJLIW	0.339769	MERBUG	0.082423	MEZLIO	0.111775	MICQIB	0.144770
MIDLIV	0.174476	MIJFES	0.585935	MIJFOB	0.165554	MILMID	0.376105
MIMDPT	0.266016	MIPDOG	0.255704	MIRWAO	0.129466	MIVSIV	0.069504
MIVTES	0.152172	MIYNUE	0.107200	MLAMPT	0.553457	MODXUB	0.424931
MOHGUN	0.545605	MOQVUJ	0.076715	MOSFAB	0.203595	MOSFEF	0.305234
MOYJAO	0.226154	MOZVOP	0.150331	MUBKIG	0.073794	MUCGAS	0.062697
MUDQOT	0.539504	MUDQUZ	0.545304	MUFRIR	0.100842	MUHQUC	0.376933
MUMZUS	0.199965	MUSXUT	0.109495	MUYHOD	0.139241	NADBID	0.086720
NAJKOY	0.082961	NAMLIW	0.043753	NANKUL	0.480235	NAPNOL	0.254747
NAPNUR	0.606902	NAPPAZ	0.168017	NAPPED	0.211668	NAQMIB	0.257945
NAQNOI	0.098428	NARCEQ	0.165494	NAVBOC	0.033144	NAVBUI	0.162117
NAVMUS	0.253688	NAYFOJ	0.140489	NECJEN	0.178393	NECKAL	0.190308
NEFXAY	0.375565	NEJWOR	0.038796	NEKYAD	0.117559	NEKZIO	0.104950
NEMDAL	0.076691	NEMGES	0.549835	NEMMAX	0.223394	NEMMUR	0.276841
NEMWEK	0.221245	NEPJAX	0.141775	NERHIC	0.193801	NERPUW	0.197931
NEVTAK	0.107243	NEVTIS	0.163863	NEZBIH	0.214253	NIFQUQ	0.416013
NIJBOZ	0.088367	NIKKEZ	0.202983	NINKOM	0.065677	NIWPOA	0.193915
NIXMIQ	0.275320	NIYBAY	0.108906	NIZMOB	0.450027	NOFTUX	0.055267
NOHPEH	0.150451	NOHPIL	0.348298	NOQLUD	0.144223	NOTSUK	0.417969
NOTZOL	0.234905	NOVDAD	0.102414	NOVDEH	0.093234	NOYBIP	0.804739
NOYFAL	0.154266	NOYXOO	0.080801	NUCVEM	0.166924	NUDBOD	0.231615
NUMCOO	0.478347	NUMCUT	0.173203	NUMCUU	0.555055	NUPWOK	1.000992
NUPWUQ	0.439851	NURBOT	0.344270	NUWFIV	0.185343	NUWFOB	0.109959
NUWFUH	0.323393	NUXNOK	0.197923	NUZBOZ	0.324954	NUZFAS	0.090478
OBEJAJ	0.135268	OBERIW	0.169185	OBEROC	0.092643	OBOPEB	0.138564
OBUQIM	0.124510	OCOJOI	0.172763	OCOJUO	0.169599	OCUBIY	0.111545
ODIFAJ	0.227401	OFAPIX	0.102148	OGOMUT	0.332519	OHEVUS	0.433370
OHIDUG	0.521409	OHIFUI	0.125173	OHIMEX	0.137582	OHOKEC	0.062242
OHOKIG	0.114899	OJABIL	0.107484	OJAGEL	0.089041	OJAGUB	0.109181
OJATAV	0.400094	OJIXAH	0.073414	OJIYOV	0.080052	OJOMAB	0.227265
OKOLEI	0.181751	OLUXIC	0.291339	OMAFOX	0.134812	OMAGAK	0.354414
OMAGEO	0.754000	OMEXUC	0.180457	ONAXOT	0.055051	ONAYAG	0.123086
ONIQIM	0.324278	ONIWEP	0.157134	ORAREF01	0.154091	OSAVOW	0.129344
OVEXEV	0.129053	OWEQOZ	0.299316	OWOZUW	0.544571	OWUBAL02	0.060621
OWUBEP02	0.405427	OXAFID	0.088549	OXOHIT	0.315906	OXUYUC	0.164747
OZAGUT	0.454636	OZAHAA	0.071910	OZUMON	0.141025	PADDAD	0.198137
PAGQAQ	0.051664	PAKYUV	0.133430	PAMFAO	0.132342	PAMFES	0.376880
PAMFIW	0.137332	PARZUD	0.090362	PAWWIU	0.178418	PAWWUG	0.140899
PAYHUW	0.328095	PAYJAE	0.144432	PECMAL	0.198225	PECMES	0.068119
PEPSEK	0.442647	PEQZES	0.204709	PEQZIW	0.078333	PERDIZ02	0.087902
PERPAF	0.225333	PERYEQ	0.094318	PEXFIL	0.181437	PEZKUC	0.056270
PIFGIU	0.085609	PIGWOT	0.326627	PIKLIG	0.208736	PIKLUS	0.353931
PILMUS	0.205577	PIQBIC	0.064449	PIXKUD	0.160699	PIXZAX	0.560409
PLPTCL	0.260226	POCYOY	0.226878	POCZAL	0.197063	POCZEP	0.214926
POJXOB	0.181292	PORXAW	0.389663	POSBUX	0.168480	POZNIC	0.225454
POZNOI	0.142407	PTMGLO01	0.492077	PUFQEM	0.113094	PUFQOW	0.115649
PUFQUC	0.130803	PUHFIJ	0.240131	PUHFUV	0.410976	PUHLUA	0.203767
PUMFIN	0.074577	PUMYAX	0.348178	PUNGIP	0.346106	PUPDUA	0.386647
PUPFEO	0.221892	PUPWOM	0.305345	PUSXEH	0.076982	PUXTAG	0.166370
PYCXPT10	0.054955	QABGEJ	0.174488	QACNOY	0.309122	QAFMAL	0.544445
QAHPUM	0.131933	QAJSAY	0.160345	QALVOO	0.305181	QAQCAM	0.125473

QASKEB	0.224134	QASKUR	0.168094	QECBAD	0.128576	QEHDAI	0.355848
QEKCEQ	0.336401	QEKCOA	0.558584	QEWZIC	0.298759	QIBJUH	0.155105
QICLOE	0.108209	QIGRAB	0.447621	QIJGAU	0.778971	QIKQAF	0.141416
QILFAS	0.115871	QIRLUY	0.153683	QITGEH	0.056815	QIVFIL	0.546943
QIXPIX	0.167536	QOCZAJ	0.147187	QODXEM	0.153157	QOGBAQ	0.161618
QOGBEU	0.251955	QOLKUY	0.066921	QOMXAR	0.111394	QORDEG	0.104023
QUBGAY	0.249342	QUBYOD	0.431210	QUGGEH	0.223894	QUNGEO	0.115588
QUNZOR	0.208971	QURKAS	0.351446	QURKUL	0.748203	QUVZOX	0.162636
QUVZUD	0.145352	QUWBEQ	0.140780	QUZPAF	0.205740	RAGHUC	0.324918
RAGJAK	0.565935	RAHNUM	0.158932	RAHSIE	0.542869	RAHTUR	0.076284
RAJKIY	0.108980	RAMZOV	0.148334	RANSOP	0.234058	RAQYUE	0.310956
RARTEM	0.074142	RATZER	0.124983	RAYRIT	0.221399	RECQIZ	0.189819
REGFEQ	0.831826	REJJAS	0.180532	REJJEW	0.203747	REJJIA	0.253861
REJVAF	0.080330	REJYAI	0.402664	RELGEV	0.194757	RERHED	0.456949
REVMAJ	0.199286	REYHOS	0.058807	RIDMOI	0.136518	RIFKUN	0.091393
RIFLAU	0.288306	RINNEH	0.121634	RINNIL	0.054939	RINNOR	0.082505
RITRAO	0.136175	RITVOH	0.078251	RITZOK	0.417240	RIVGAG	0.183371
RIVGEK	0.151041	RIVGIO	0.438247	RIVJOV	0.075043	RIVNUH	0.067923
RIVVAU	0.253487	RIVVEY	0.429048	RIYJUF	0.072511	ROGYIW	0.252096
ROKJOQ	0.035325	ROLXOG	0.155891	ROMLIO	0.202457	ROMQUH	0.329324
RUJPIV	0.083988	RUJRAP	0.282171	RULKOZ	0.344428	RUNJIU	0.197365
RUNJOA	0.198039	RUPXUW	0.120553	RUTMEY	0.058893	RUVKOK	0.381233
SADHIS	0.194121	SAGLAR	0.312806	SAGLIZ	0.233147	SAHLIZ	0.075450
SAKNAU	0.406349	SATSIR	0.186075	SEDQOK	0.100255	SEHCEO	0.146638
SEKNAY	0.113316	SEMTUA	0.400198	SEMXEQ	0.091897	SERDOJ	0.098996
SERPIP	0.279818	SESDOJ	0.285147	SESTUH	0.242591	SESXIX01	0.282088
SETWEX	0.104120	SEVNUF	0.167430	SIBSEB	0.509349	SICMUO	0.134817
SICNEZ	0.086917	SIMFEB	0.361499	SIMFOL	0.235152	SISMIQ	0.339093
SISWIA	0.184572	SIWXIH	0.151995	SIWXON	0.169288	SIXLEQ	0.108425
SOBPUW	0.081483	SOFYOB	0.666834	SOKVAP	0.086865	SOLBOK	0.252652
SOLCAX	0.113852	SONNEO	0.046462	SOPQOE	0.107755	SORVUR	0.434276
SOSTAX	0.055689	SOSVIH	0.323948	SOTXAB	0.202733	SOVZUZ	0.443952
SOWBAI	0.090744	SOWTED	0.152892	SOYSUU	0.133908	SUCNOV	0.096845
SUCPEL	0.119965	SUDJAF	0.316240	SUDJIN	0.160581	SUDMIN02	0.139488
SUJFAH	0.088478	SUKMIW	0.524626	SUQKOG	0.440828	SUQLIB	0.190628
SURQAY	0.079242	SUSSEG	0.073391	SUSSEG01	0.075250	SUTSUV	0.245011
TAGHIT	0.069262	TAGHOZ	0.119840	TAJTED	0.096421	TANDOC	0.451178
TAQMED	0.051422	TARFAT	0.109485	TASNOS	0.361612	TAXKIN	0.110591
TAXTIW	0.290197	TAYJEM	0.113457	TAYYIC	0.158803	TAZRIX	0.302613
TCAMPT	0.384041	TEBNEW	0.108298	TECWEE	0.276252	TEFPOK	0.179905
TEGYAG	0.144781	TEJYAJ	0.364968	TELGOG	0.171923	TELNOQ	0.132589
TELQEI	0.078255	TEMPUA	0.104349	TEMQEL	0.076974	TEPREN	0.306866
TEQTOZ	0.114263	TERZUL	0.304911	TESYID	0.106394	TETVOD	0.205483
TEVSAP	0.167360	TIBZEJ	0.306865	TIHVEM	0.099641	TIHVIQ	0.105791
TIHYIU	0.122130	TINSEP	0.423941	TIQHUY	0.498452	TIRDIJ	0.164028
TIZNOG	0.093560	TIZWEE	0.278450	TMAMPT01	0.028463	TOBFOF	0.390154
TOBQOR	0.158407	TODMEG	0.797858	TODMIK	0.419559	TOGFEC	0.499268
TOGJAC	0.264065	TOGJOQ	0.407561	TOMSIZ	0.397136	TOPVUQ	0.482806
TOPWAX	0.670477	TOQKEQ	0.328248	TOQPEW	0.071497	TORJIT	0.423793
TOXHAP	0.150366	TOXQUS	0.076072	TOXYAG	0.090895	TUCJIL	0.173203
TUKWIF	0.129636	TUNQAW	0.085535	TURJEW	0.286797	TUWTIO	0.105377
UBECOT	0.192147	UBEDEK	0.146995	UBIXIM	0.098619	UCIZUC	0.153191
UCOBEU	0.096660	UFASOJ	0.065272	UFASUP	0.344712	UFETIK	0.086835
UGETEG	0.144158	UGOQAL	0.143799	UHEDAN	0.284125	UHOCOJ	0.125018
UHUPUI	0.086070	UHUYIG	0.180632	UHUZEC	0.200160	UJEKUP	0.081735
UKASUU	0.247284	UKAYUB	0.322303	UKECIW01	0.361400	UKECIW	0.306981
UKUGOW	0.106619	ULIFAZ	0.223559	ULUTOL	0.291775	UMACET	0.331920
UMOBEE	0.229509	UMOBII	0.226809	USAFUS	0.369721	USAGED	0.240400
USIROE	0.071960	USIVAV	0.024505	USOWOP	0.083756	UTAWOC	0.246685
UWOROQ	0.075994	UXEDUZ	0.124293	UXEVOL	0.110216	UYANEO	0.233399
UZOCAQ	0.130765	UZOCIY	0.185269	VAMTUB	0.276372	VAMYEP	0.176484
VAMYUF	0.131588	VANJID	0.278330	VANKUQ	0.052341	VAPXOB	0.173682
VAQVIT	0.340853	VAYGEI	0.069897	VAYHEL	0.180209	VAYHOV	0.193817
VAYHUB	0.390157	VAYXEY	0.292885	VAZWEY	0.371635	VEDZAG	0.251239
VEGCUG	0.122577	VEGDER	0.112531	VEGVIN	0.164772	VEJVAK	0.220191
VEKFIB	0.475580	VEKFUO	0.183612	VENHAA01	0.297748	VEQLIO	0.163166
VERYEW	0.160460	VETGIK	0.150314	VEYDOT	0.408830	VEZHOA	0.120150
VEZJOC	0.122698	VIBYOV	0.173227	VIDGIA	0.225177	VIKPIP	0.383270
VILJAB	0.167307	VILJOR	0.170839	VINPAC	0.108122	VISHEM	0.335067
VITXOL01	0.445002	VITYON	0.229614	VITZOO	0.631914	VOCLEF	0.066923

VOCLIJ	0.092132	VOCLOP	0.434385	VOHBAW	0.290929	VOPFIP	0.113548
VOPLAP	0.109668	VOZYUE	0.174917	VUCNOX	0.152365	VUKSUS	0.294663
VUKYOP	0.427087	VUPMEA	0.177506	VUPMIE	0.270167	VUTRIL	0.181377
VUTYIV	0.056875	VUXREO	0.438660	VUZDOK	0.199705	WAGNOJ	0.302855
WAJVOS	0.196565	WAJYAK	0.195339	WAKTIP	0.148115	WALXUE	0.233373
WAPFUS	0.168123	WAQHAX	0.104672	WAQWAM	0.453276	WARTIU	0.197511
WEDJEU	0.080849	WEGHEZ	0.651755	WEGSUX	0.219722	WEHNII	0.125624
WEHTUC	0.098505	WENQUC	0.078340	WERCIF	0.274154	WERZIC	0.599380
WESQUH	0.301807	WESRAO	0.161685	WESXOK	0.077249	WESZED	0.065587
WEXFIQ	0.062782	WEYNIA	0.088696	WEZWIH	0.035532	WICQUU	0.309138
WIFPEJ	0.109669	WIFPIN	0.150071	WIFQIO	0.309980	WIHMOR	0.819022
WINCOO	0.268227	WIRJAI	0.154437	WIRRIA	0.409400	WIRROG	0.204346
WIRRUM	0.343925	WIYXUX	0.120865	WOCKOQ	0.503385	WODCIB	0.415305
WODGIH	0.163221	WOJGAE	0.127850	WOVKUP	0.286313	WOYGEW	0.216106
WOZXAK	0.233100	WUFDOQ	0.089208	WUFFUY	0.074026	WUFKAJ	0.396883
WUFKEN	0.444300	WUFKIR	0.222828	WUFKUD	0.197392	WUFLAK	0.240951
WUJKIV	0.196460	WUJYAE	0.131835	WUJYOS	0.226602	WUQNOL	0.340011
WURGAU	0.082245	WURNAA	0.383480	WURNEE	0.335060	WURROS	0.121487
WUXCEA	0.032957	WUZSAL	0.153832	XABGOA	0.146993	XAHJAV	0.461726
XAHJID	0.237652	XAHJOJ	0.220319	XAJWOW	0.046550	XAKBEQ	0.325203
XAKPAA	0.337410	XARDOK	0.265860	XARFEC	0.171531	XARFIG	0.143023
XARFOM	0.134391	XATYEX	0.103906	XAVMIR	0.083719	XAVRIW	0.204501
XAWLOZ	0.103430	XAWLUF	0.068430	XAWWID	0.226682	XAWWOJ	0.135393
XEMVOE	0.535893	XENYIZ	0.169679	XERZOL	0.174056	XESPIV	0.402703
XESPOB	0.204180	XETFUZ	0.454034	XETGIM	0.410241	XETGOS	0.172222
XEXMUK	0.120529	XEZXOR	0.185213	XIDQUX	0.495792	XIDRAE	0.395155
XIDREI	0.311858	XILMOV	0.011864	XIMGEG	0.102876	XINTOF	0.088338
XIPJOV	0.148673	XITYEH	0.119729	XITYIL	0.115773	XIVDEL	0.187091
XIXTIJ	0.085752	XIXTUV	0.168585	XIXVAD	0.095259	XIXVEH	0.086151
XIXVIL	0.086426	XIYJEW	0.073048	XOBKIK	0.085514	XOCNOV	0.172769
XONKAP	0.141244	XOVFIZ	0.102383	XOVFUJ	0.415846	XUGZAD	0.074595
XUHMOC	0.675828	XUHNAP	0.374542	XUKWUV01	0.098995	XUKWUV	0.197948
XULFEP	0.117616	XULVUY	0.705286	XUPBIT	0.282443	XUPMOM	0.321361
XUVKEF	0.475398	XUXJUV	0.522121	XUXKAC	0.240495	YABJAN	0.128412
YACLEV	0.463859	YACLOF	0.266148	YAGBEN	0.296194	YAGQIG	0.065062
YAJDUJ	0.522542	YAPZEW	0.309670	YASPIR	0.129839	YASPOX	0.154036
YATPER	0.064706	YATTAO	0.198786	YEFJAW	0.368867	YEJWUG	0.182467
YEJXAN	0.189962	YEKHOM	0.200275	YEQSIX	0.095661	YEQSOD	0.127411
YEQSUJ	0.211304	YEXDEM	0.165494	YIBWAJ	0.163484	YIBWEN	0.484368
YIJBUR	0.306277	YIRKOZ	0.524052	YIRKUF	0.094233	YIYVEK	0.251326
YOBGII	0.124256	YODBEY01	0.114468	YODBIC01	0.088442	YODFOM	0.064478
YOKCOR	0.100455	YONWIJ	0.110193	YOPNEW	0.256933	YOVZOZ	0.083843
YOWHIB	0.401122	YUBJAG	0.058891	YUBJEK	0.458475	YUDBOP	0.359940
YUDBUU	0.148267	YUHRUO	0.245637	YUMROO	0.066418	YUXKEK	0.107976
ZADTUW	0.071038	ZAHJAT	0.087509	ZARVOH	0.376650	ZATZUP	0.629097
ZAVHIQ	0.339305	ZAVXOL	0.152115	ZEBNOJ	0.054568	ZECLUR	0.342245
ZEDREI	0.328823	ZEGQAF	0.339087	ZEHPEJ	0.032876	ZEKLEJ	0.262130
ZEKLIN	0.132416	ZENDAA	0.167291	ZENDII	0.106955	ZEPTIZ	0.094958
ZEZTEF	0.374020	ZEZTIJ	0.157026	ZIBKUS	0.116127	ZIBLED	0.141781
ZOSSOS	0.491222	ZOTMEA	0.140084	ZOVLOL	0.177058	ZUGSID	0.130835
ZULVAD	0.163424	ZUNTOT	0.215521	ZURBOD	0.472560	ZUTHOL	0.225164
ZUTREN	0.437169	ZUYWEX	0.191811	ZUYZIF	0.473526	ZUZBAA	0.169581
ZUZNOA	0.162687	ZUZTIX	0.199975	ZZZFCW01	0.076859		

References

[1] Michael Gaus, Albrecht Goez, and Marcus Elstner. Parametrization and benchmark of DFTB3 for organic molecules. *J. Chem. Theory Comput.*, 9:338–354, 2012.

[2] Xiya Lu, Michael Gaus, Marcus Elstner, and Qiang Cui. Parametrization of dftb3/3ob for magnesium and zinc for chemical and biological applications. *The J. Phys. Chem. B*, 119:1062–1082, 2014.

[3] M. Gaus, X. Lu, M. Elstner, and Q. Cui. Parameterization of DFTB3/3OB for sulfur and phosphorus for chemical and biological applications. *J. Chem. Theory Comput.*, 10:1518–1537, 2014.

[4] M. Kubillus, T. Kubař, M. Gaus, Jan Řezáč, and M. Elstner. Parameterization of the DFTB3 method for Br, Ca, Cl, F, I, K, and Na in organic and biological systems. *J. Chem. Theory Comput.*, 11:332–342, 2014.

[5] Maxime Van den Bossche. DFTB-assisted global structure optimization of 13- and 55-atom late transition metal clusters. *J. Phys. Chem. A*, 123:3038–3045, 2019.

[6] Maxime Van den Bossche. Three-center tight-binding together with multipolar auxiliary functions. *J. Chem. Theory Comput.*, 20:2538–2550, 2024.

[7] Maxime Van den Bossche, Henrik Groenbeck, and Bjork Hammer. Tight-binding approximation-enhanced global optimization. *J. Chem. Theory Comput.*, 14:2797–2807, 2018.

[8] Ask Hjorth Larsen, Jens Jørgen Mortensen, Jakob Blomqvist, Ivano E Castelli, Rune Christensen, Marcin Dułak, Jesper Friis, Michael N Groves, Bjørk Hammer, Cory Hargus, Eric D Hermes, Paul C Jennings, Peter Bjerre Jensen, James Kermode, John R Kitchin, Esben Leonhard Kolsbjerg, Joseph Kubal, Kristen Kaasbjerg, Steen Lysgaard, Jón Bergmann Maronsson, Tristan Maxson, Thomas Olsen, Lars Pastewka, Andrew Peterson, Carsten Rostgaard, Jakob Schiøtz, Ole Schütt, Mikkel Strange, Kristian S Thygesen, Tejs Vegge, Lasse Vilhelmsen, Michael Walter, Zhenhua Zeng, and Karsten W Jacobsen. The atomic simulation environment—a python library for working with atoms. *J. Physics: Condens. Matter*, 29:273002, 2017.
